# Fragile X mental retardation protein controls synaptic vesicle exocytosis by modulating N-type calcium channel density

**DOI:** 10.1038/ncomms4628

**Published:** 2014-04-07

**Authors:** Laurent Ferron, Manuela Nieto-Rostro, John S. Cassidy, Annette C. Dolphin

**Affiliations:** 1 Department of Neuroscience, Physiology and Pharmacology, University College London, Gower Street, London WC1E 6BT, UK; 2 These authors contributed equally to this work

## Abstract

Fragile X syndrome (FXS), the most common heritable form of mental retardation, is characterized by synaptic dysfunction. Synaptic transmission depends critically on presynaptic calcium entry via voltage-gated calcium (Ca_V_) channels. Here we show that the functional expression of neuronal N-type Ca_V_ channels (Ca_V_2.2) is regulated by fragile X mental retardation protein (FMRP). We find that FMRP knockdown in dorsal root ganglion neurons increases Ca_V_ channel density in somata and in presynaptic terminals. We then show that FMRP controls Ca_V_2.2 surface expression by targeting the channels to the proteasome for degradation. The interaction between FMRP and Ca_V_2.2 occurs between the carboxy-terminal domain of FMRP and domains of Ca_V_2.2 known to interact with the neurotransmitter release machinery. Finally, we show that FMRP controls synaptic exocytosis via Ca_V_2.2 channels. Our data indicate that FMRP is a potent regulator of presynaptic activity, and its loss is likely to contribute to synaptic dysfunction in FXS.

## Abstract (web summary)

Mutations in the fragile X mental retardation protein are implicated in synaptic dysfunction in fragile X syndrome. Here, Ferron *et al*. show that fragile X mental retardation protein maintains proper neurotransmission by regulating the density of N-type calcium channels in the presynaptic terminal.

## Graphical abstract

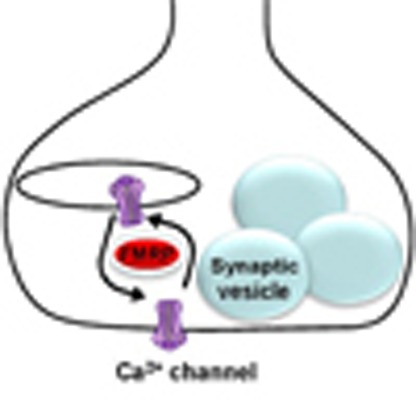

Fragile X syndrome (FXS) is the most common inherited form of intellectual disability, often associated with autism^1^,^2^. FXS is caused, in the vast majority of cases, by a trinucleotide repeat expansion in the 5′-UTR of the fragile X mental retardation 1 (*FMR1*) gene, leading to its hypermethylation and transcriptional silencing. The *FMR1* gene encodes fragile X mental retardation protein (FMRP), an RNA-binding protein that participates in the trafficking of messenger RNA (mRNAs) to distal sites in neurons, especially dendrites of central neurons^3^. FMRP suppresses translation of mRNAs to which it binds, but also regulates the activity-dependent translation of these mRNAs, particularly in response to group I metabotropic glutamate receptor activation^4^,^5^. These findings show that dendritic FMRP is critical for postsynaptic activity of neurons^6^.

A growing body of evidence points to an additional role for FMRP in presynaptic function^7^,^8^,^9^. For example, a recent study showed that *Fmr1* knockout mice have abnormal presynaptic short-term plasticity in hippocampal neurons^10^. This opened the possibility that there is an effect of FMRP on presynaptic voltage-gated calcium (Ca_V_) channels.

The Ca_V_ family plays a major role in the physiology of excitable cells^11^. Three subfamilies have been identified: Ca_V_1–3. The Ca_V_1 (L-type) and Ca_V_2 (N-, P/Q- and R-type) channels are thought to be heteromultimers composed of the pore-forming α_1_-subunit, associated with auxiliary Ca_V_β- and Ca_V_α_2_δ-subunits^11^. N-type calcium channels (Ca_V_2.2) are present in both the central and peripheral nervous systems, and they have a major presynaptic role in regulation of transmitter release^12^,^13^. The intracellular loop between domains II and III and the C-terminal tail of Ca_V_2 α_1_-subunits interact with presynaptic proteins that modulate calcium currents and the targeting of these channels to synaptic terminals^14^. Since there are precedents for interaction of FMRP with ion channels, specifically the sodium-activated K^+^ channel (Slack-B)^15^ and the calcium-activated potassium channel (BK)^16^, we examined whether FMRP could directly affect calcium channel function.

Here we show that Ca_V_ channel density in dorsal root ganglion (DRG) neurons is increased at the cell surface of the soma and the presynaptic terminals when FMRP is knocked down with small hairpin RNA (shRNA). We then show that FMRP reduces Ca_V_2.2-generated currents by decreasing the expression of the channel at the plasma membrane, and we provide evidence that this mechanism involves proteasomal degradation. We also show that the interaction between FMRP and Ca_V_2.2 is direct, since we find that the C-terminal domain of FMRP binds to the Ca_V_2.2 II–III linker and C-terminal domains. Finally, using a pharmacological approach, we show that FMRP modulates synaptic vesicle release via Ca_V_2.2 channels. Our data demonstrate an unexpected effect of FMRP on Ca_V_2.2 channel functional expression, and indicate a new role for FMRP in presynaptic function.

## Results

### FMRP regulates Ca_V_2.2 expression in DRG neurons

**Figure 1:**
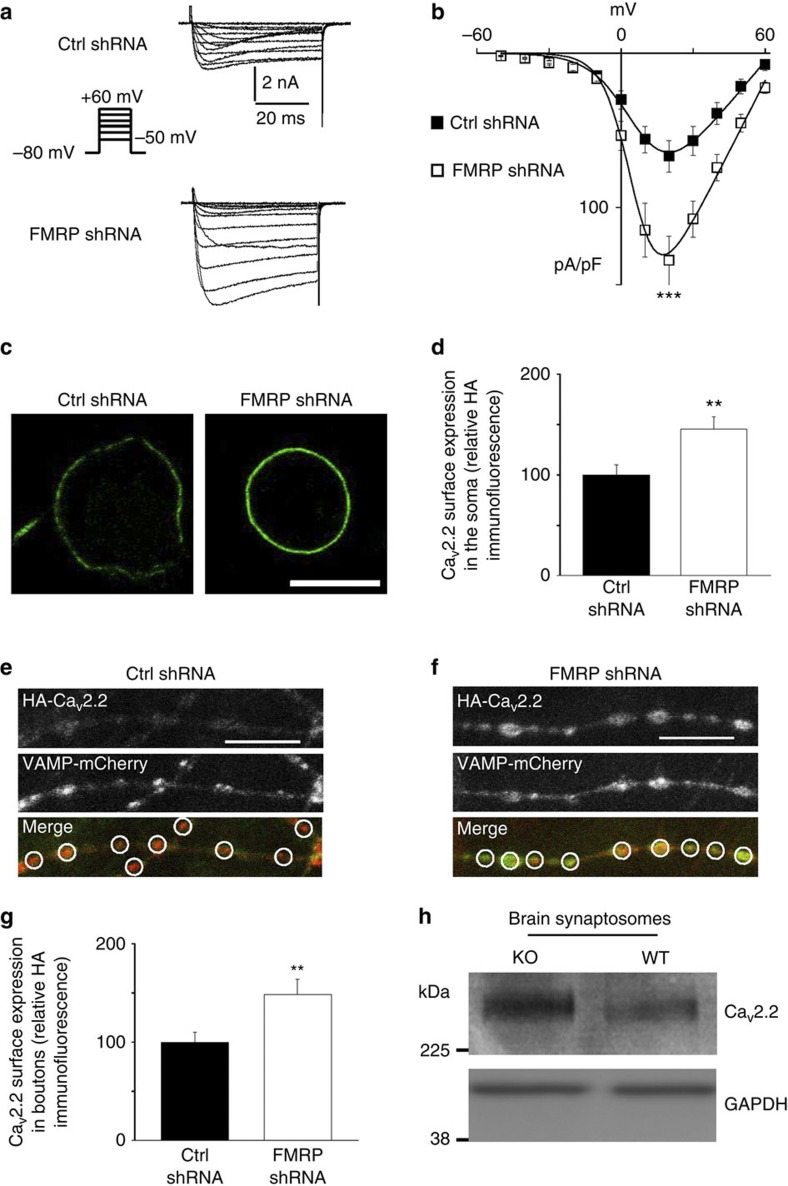
FMRP knockdown increases Ca_V_ currents and enhances surface expression of Ca_V_2.2 channels in soma and presynaptic terminals of DRG neurons. (**a**) Typical calcium channel current traces recorded from DRG neurons transfected with Ctrl shRNA (top) or FMRP shRNA (bottom), elicited by 50 ms step depolarizations between −50 and +60 mV from a holding potential (HP) of −80 mV. The charge carrier was 10 mM Ba^2+^. (**b**) Current–voltage relationship for calcium channel current (*I*_Ba_) recorded from DRG neurons transfected with Ctrl shRNA (filled squares, *n*=16) or FMRP shRNA (open squares, *n*=14). Peak currents were normalized to the cell capacitance. For +20 mV, *I*_Ba_ was −66.7±10.3 pA/pF in Ctrl shRNA (*n*=16) and −134.3±15.8 pA/pF in FMRP shRNA (*n*=14, *P*=0.001). The mean data are fitted with a modified Boltzmann function (see Methods) with *V*_50,act_ of +7.9±1.6 and +5.8±0.8 mV, respectively, and *G*_max_ of 1.9±0.2 and 3.1±0.2 nS/pF, respectively. Means±s.e.m., ***P<0.001; one-way ANOVA. (**c**) Representative confocal images of HA staining from non-permeabilized DRG neurons expressing HA-Ca_V_2.2/Ca_V_β1b/Ca_V_α_2_δ-1 with Ctrl shRNA (left) or FMRP shRNA (right). Scale bar, 20 μm. (**d**) Bar chart showing normalized cell surface expression of HA-Ca_V_2.2 in DRG neurons expressing Ctrl shRNA (filled bar, 100±10%, *n*=51 cells) or FMRP shRNA (open bar, 145±12%, *n*=62 cells, *P*=0.006). Means±s.e.m., **P<0.01; one-way ANOVA. (**e**,**f**) Representative confocal images of non-permeabilized DRG neuron processes expressing HA-Ca_V_2.2/Ca_V_β1b/Ca_V_α_2_δ-1/VAMP-mCherry with Ctrl shRNA (**e**) or FMRP shRNA (**f**). Top panels show HA-Ca_V_2.2 immunostaining, middle panels show VAMP-mCherry and bottom panels show merged HA-Ca_V_2.2 immunostaining (green) with presynaptic VAMP-mCherry (red). Bouton regions are indicated by the white circles. Scale bars, 10 μm. (**g**) Bar chart showing normalized cell surface expression of HA-Ca_V_2.2 in boutons of DRG neurons expressing Ctrl shRNA (filled bar, 100±10%, *n*=73) and FMRP shRNA (open bar, 148±15%, *n*=50, *P*=0.006). Means±s.e.m., **P<0.01; one-way ANOVA. (**h**) Immunoblotting for Ca_V_2.2 in brain synaptosomes from *Fmr1* knockout (KO) and wild-type (WT) mice. Glyceraldehyde 3-phosphate dehydrogenase (GAPDH) provides a loading control for Ca_V_2.2 quantification. Ca_V_2.2 expression in brain synaptosomes from *Fmr1* knockout mice is increased by 43% (average of two independent experiments, 38 and 48%). Full-size blots can be found in Supplementary Fig. 8.

To determine the impact of FMRP on Ca_V_ currents in DRG neurons, we silenced FMRP expression using RNA interference. Knockdown of FMRP was validated in tsA-201 cells expressing green fluorescent protein (GFP)-FMRP and in cultured DRG neurons (Supplementary Fig. 1). We then examined the effect of FMRP knockdown on calcium currents in DRG neurons 4 days after shRNA transfection (Fig. 1a,b). We found that, in DRG neurons in which FMRP was knocked down, the peak Ca_V_ current density was increased by 103% (Fig. 1b). Previous work found that N-type calcium currents account for the major component of calcium current in DRG neurons^17^. We then examined the impact of FMRP knockdown on Ca_V_2.2 surface expression. To do this, we transfected DRG neurons with a Ca_V_2.2 channel construct tagged with an exofacial HA epitope and either control shRNA or FMRP shRNA (Fig. 1c). We found that HA-Ca_V_2.2 immunoreactivity at the cell surface was increased by 45% in neurons in which FMRP was knocked down (Fig. 1c,d).

We then took advantage of the fact that DRG neurons form functional synapses, when co-cultured with dorsal horn (DH) neurons^18^, to investigate the impact of FMRP on Ca_V_2.2 expression at presynaptic terminals. P2 DRG neurons were co-microinjected with HA-Ca_V_2.2 and the presynaptic protein VAMP-1, tagged with mCherry, together with either control shRNA or FMRP shRNA (Fig. 1e,f). We found that HA-Ca_V_2.2 immunoreactivity in VAMP-mCherry-positive boutons in non-permeabilized processes was increased by 48% in neurons in which FMRP was knocked down (Fig. 1g). Finally, we assessed Ca_V_2.2 expression in brain synaptosomes from *Fmr1* knockout mice (Fig. 1h). We found that the level of Ca_V_2.2 protein in synaptosomes was increased by 43% in *Fmr1* knockout mice compared with wild-type mice. Together, our data reveal that FMRP controls the presynaptic expression of Ca_V_2.2 in DRG neurons.

### FMRP controls Ca_V_2.2 current density

**Figure 2:**
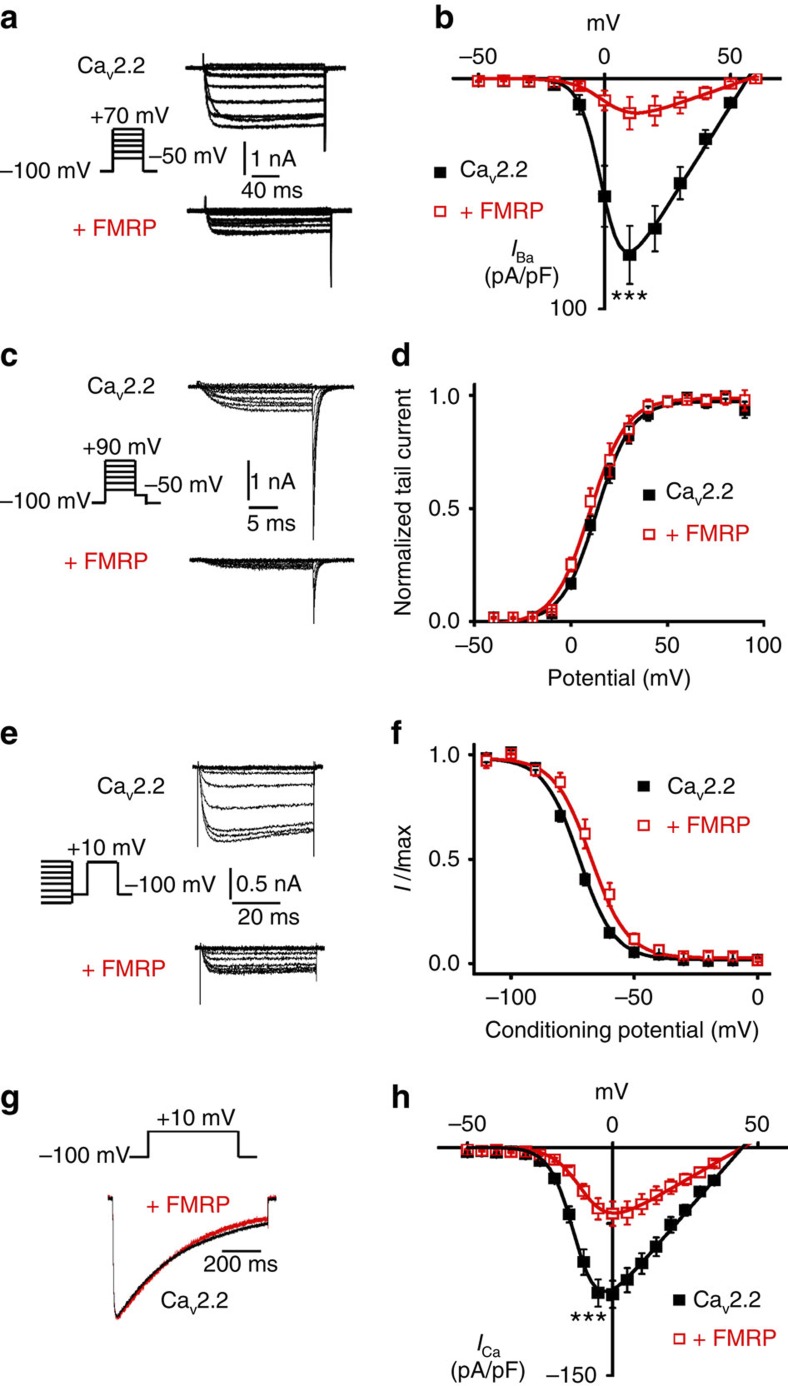
FMRP reduces Ca_V_2.2 current density. Recordings were made from tsA-201 cells transfected with Ca_V_2.2/Ca_V_β1b/Ca_V_α_2_δ-1, with or without GFP-FMRP. (**a**) Ca_V_2.2 current traces elicited by 100 ms step depolarizations between −50 and +70 mV from a HP of −100 mV, for Ca_V_2.2 (top) and Ca_V_2.2+FMRP (bottom). The charge carrier was 1 mM Ba^2+^. (**b**) Current–voltage relationship obtained with Ca_V_2.2 (filled squares, *n*=5) and Ca_V_2.2+FMRP (red open squares, *n*=8). *V*_50,act_=−0.4±0.4 and 1.4±1.2 mV, respectively, and *G*_max_=1.7±0.1 and 0.4±0.1 nS/pF, respectively. At +10 mV, peak Ca_V_2.2 *I*_Ba_ current density were −76.5±12.6 pA/pF (*n*=5) and −15.1±7.1 pA/pF (*n*=8, *P*=0.007) for Ca_V_2.2 and Ca_V_2.2+FMRP, respectively (means±s.e.m., ***P<0.001; one-way ANOVA). (**c**) Current traces illustrating current activation for Ca_V_2.2 (top) and Ca_V_2.2+FMRP (bottom). Peak tail currents were recorded after repolarization to −50 mV after a 20 ms test pulse between −50 and +90 mV from a HP of −100 mV. (**d**) Voltage dependence of activation for Ca_V_2.2 (filled squares) and Ca_V_2.2+FMRP (red open squares). *V*_50_=+13.8±1.8 mV for Ca_V_2.2 and +11.2±2.8 mV for Ca_V_2.2+FMRP (*n*=6; means±s.e.m., *P*=0.444; one-way ANOVA). (**e**) Current traces illustrating steady-state inactivation for Ca_V_2.2 (top) and Ca_V_2.2+FMRP (bottom). Ba^2+^ currents were recorded after conditioning pulses of 10-s duration, applied from a HP of −100 mV in 10 mV steps between −110 to 0 mV, followed by a 50 ms test pulse to +10 mV. (**f**) Voltage dependence of steady-state inactivation for Ca_V_2.2 (filled squares) and Ca_V_2.2+FMRP (red open squares). V_50,inact_=−74.3±1.2 mV for Ca_V_2.2 (*n*=5) and −66.3±1.8 mV for Ca_V_2.2+FMRP (*n*=6; means±s.e.m., *P*=0.007; one-way ANOVA). (**g**) Normalized current traces for Ca_V_2.2 (black) and Ca_V_2.2+FMRP (red) in response to 800 ms depolarization step to +10 mV from a HP of −100 mV. Mean time constants of inactivation (*τ*_inact_) obtained by fitting the decaying phase of the current at +10 mV with a single exponential were 228±37 ms (*n*=7) and 198±17 ms (*n*=8, means±s.e.m., *P*=0.46; one-way ANOVA) for Ca_V_2.2 and Ca_V_2.2+FMRP, respectively. (**h**) Current–voltage relationship obtained in cells transfected with Ca_V_2.2 (filled squares, *n*=7) and Ca_V_2.2+FMRP (open squares, *n*=11) using 1 mM Ca^2+^ as a charge carrier and *N*-methyl-D-glucamine in the pipette solution. *V*_50,act_=−12.4±0.4 mV and −9.7±0.4 mV, respectively, and *G*_max_=2.2±0.1 and 1.1±0.1 nS/pF, respectively. At 0 mV, peak Ca_V_2.2 current density were −96.6±8.9 pA/pF (*n*=7) and −43.7±8.0 pA/pF (*n*=11, *P*=0.0005) for Ca_V_2.2 and Ca_V_2.2+FMRP, respectively. Means±s.e.m., ***P<0.001; one-way ANOVA.

To characterize the functional effect of FMRP on N-type calcium channels, we recorded Ba^2+^ currents (*I*_Ba_) from tsA-201 cells co-transfected with Ca_V_2.2 (together with auxiliary subunits Ca_V_β1b and Ca_V_α_2_δ-1) and GFP-FMRP. We found that FMRP significantly reduced peak Ca_V_2.2 *I*_Ba_ current density at +10 mV by 80% (Fig. 2a,b).

A reduction of current density can result from a loss of functional channels at the plasma membrane and/or a modification of the biophysical properties of the channels. No difference in the voltage dependence of activation for the current density–voltage relationships was observed when FMRP was co-expressed with Ca_V_2.2 (Fig. 2b) and this was confirmed by tail current analysis (Fig. 2c,d). However, we noted a consistent depolarizing shift of the Ca_V_2.2 steady-state inactivation curve when FMRP was co-expressed (Fig. 2e,f). This latter modification is not responsible for the reduction of current density but nevertheless it suggests that FMRP may be interacting with intracellular domains of Ca_V_2.2 involved in voltage-dependent inactivation. We also examined the kinetics of decay of the current, but observed no modification of the time constant of inactivation (Fig. 2g). Importantly, the reduction in current density was still evident using the physiological charge carrier, Ca^2+^; FMRP reduced peak Ca_V_2.2 I_Ca_ density at 0 mV by 55% (Fig. 2h).

**Figure 3:**
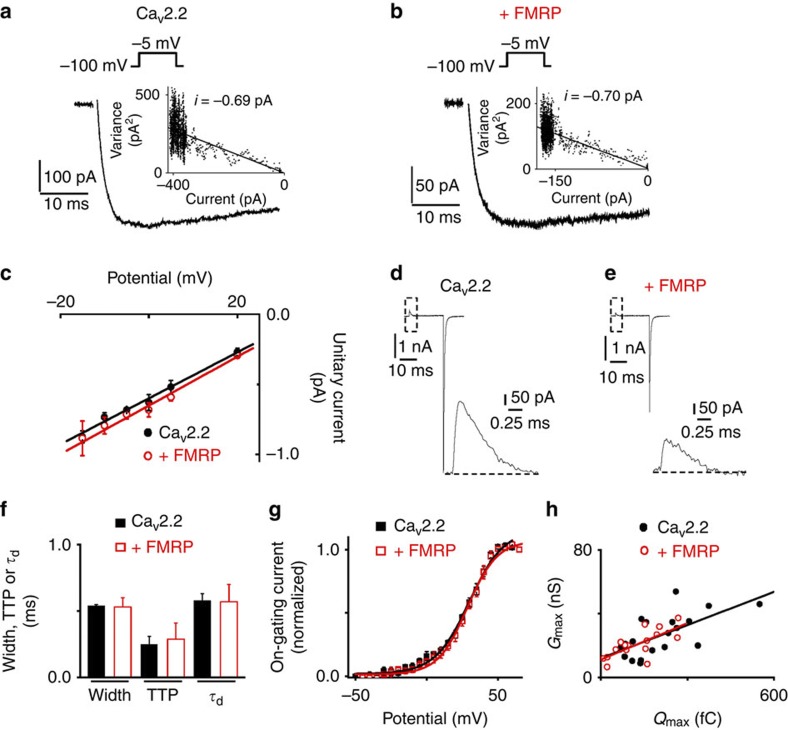
Effect of FMRP on biophysical properties of Ca_V_2.2. (**a**,**b**) Noise analysis for Ca_V_2.2 currents recorded in 10 mM Ba^2+^ from tsA-201 cells transfected with Ca_V_2.2 (**a**) or Ca_V_2.2+FMRP (**b**), plus α_2_δ-1/β1b. Average currents in response to a 50 ms step depolarization to −5 mV from −100 mV (average of 80 depolarization steps). Inset, variance-current plot. The unitary current at −5 mV, *i*, determined from the slope, is −0.69 pA for Ca_V_2.2 and −0.70 pA for Ca_V_2.2+FMRP. (**c**) Plot of unitary current estimated from noise analysis plotted against test potential for Ca_V_2.2 (filled circles) and Ca_V_2.2+FMRP (red open circles). A minimum of three test potentials were recorded per cell (3–5 cells). The slope, determined by linear regression corresponding to the single-channel conductance, was 16.4±1.1 pS and 17.4±1.2 pS for Ca_V_2.2 and Ca_V_2.2+FMRP, respectively. (**d**,**e**) Traces showing gating currents for Ca_V_2.2 and Ca_V_2.2+FMRP measured at reversal potential (average of 10 depolarization steps). Inset, ON-gating current, taken from the boxed areas. ON-gating current densities were 14.9±1.6 pA/pF (*n*=23) and 10.5±1.2 pA/pF (*n*=24) for Ca_V_2.2 and Ca_V_2.2+FMRP, respectively (means±s.e.m., *P*=0.028; one-way ANOVA). (**f**) Peak ON-gating current properties for Ca_V_2.2 (filled bars, *n*=8) and Ca_V_2.2+FMRP (red open bars, *n*=11). Width (at 50% of the maximum gating current): 0.54±0.01 ms for Ca_V_2.2 and 0.53±0.07 ms for Ca_V_2.2+FMRP (*P*=0.9); TTP (time-to-peak): 0.25±0.06 ms for Ca_V_2.2 and 0.29±0.12 ms for Ca_V_2.2+FMRP (*P*=0.3); and *τ*_d_ (decay time constant): 0.58±0.05 ms for Ca_V_2.2 and 0.57±0.13 ms for Ca_V_2.2+FMRP (*P*=0.6). Means±s.e.m., one-way ANOVA. (**g**) Voltage dependence of ON-gating current activation for Ca_V_2.2 (filled squares) and Ca_V_2.2+FMRP (red open squares). Ca_V_2.2 currents were elicited by 20 ms step depolarizations between −50 and +60 mV from −100 mV. Mean data are fitted with a Boltzmann function, *V*_50_=+29.9±1.2 mV for Ca_V_2.2 and +28.5±0.9 mV for Ca_V_2.2+FMRP (*n*=6; *P*=0.94). Means±s.e.m., one-way ANOVA. (**h**) Relationship between maximum whole-cell conductance (*G*_max_) and maximum gating charge (*Q*_max_) for Ca_V_2.2 (closed circles) and Ca_V_2.2+FMRP (red open circles). At reversal potential, *Q*_max_ was 203.4±27.4fC (*n*=20) and 125.0±20.6fC (*n*=16, *P*=0.035) for Ca_V_2.2 and Ca_V_2.2+FMRP, respectively (means±s.e.m., one-way ANOVA). Data were fitted by linear regression (Ca_V_2.2, black; Ca_V_2.2+FMRP, red). The slopes (*G*_max_/*Q*_max_), proportional to maximal channel open probability were 0.069±0.018 nS/fC (*n*=20 cells) and 0.076±0.019 nS/fC (*n*=16 cells) for Ca_V_2.2 and Ca_V_2.2+FMRP, respectively.

Since FMRP has been shown to modulate the gating of Slack-B channels^15^, we therefore assessed the impact of FMRP on Ca_V_2.2 channel activity. Using fluctuation analysis, we found that FMRP does not modify the single-channel conductance of Ca_V_2.2 (Fig. 3a–c). Alternatively, a reduction of open probability of the channels could also account for a lower whole-cell current density. We therefore estimated Ca_V_2.2 maximum open probability by comparing the maximal ON-gating charge displaced (*Q*_max_) with the whole-cell conductance (*G*_max_) for each cell^19^ (Fig. 3d–h). We found that *Q*_max_ for Ca_V_2.2 was reduced by 38% when FMRP was co-expressed, suggesting there are fewer channels in the plasma membrane; but neither the gating charge kinetics (Fig. 3f) or voltage dependence (Fig. 3g), nor the estimated channel maximum open probability (Fig. 3h) were modified. Taken together, our results indicate that FMRP is likely to act by reducing the number of available Ca_V_2.2 channels on the cell surface.

### FMRP reduces cell surface Ca_V_2.2 via proteasomal degradation

**Figure 4:**
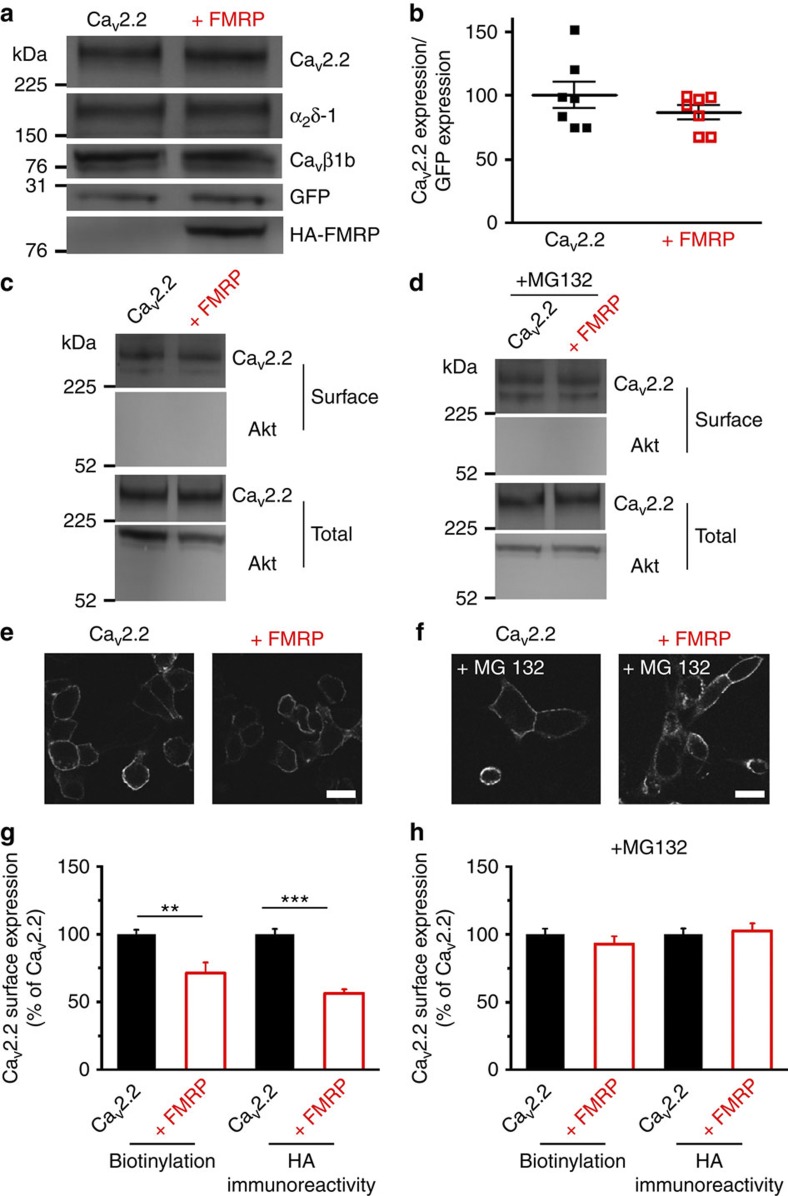
FMRP reduces Ca_V_2.2 cell surface expression. (**a**) Immunoblots of lysates from tsA-201 cells expressing Ca_V_2.2 (left) or Ca_V_2.2+FMRP (right), plus Ca_V_β1b/Ca_V_α_2_δ-1. Samples were immunoblotted with Ca_V_2.2, Ca_V_β1b, Ca_V_α_2_δ-1, GFP or HA Abs. Full-size blots can be found in Supplementary Fig. 8. (**b**) Expression of total Ca_V_2.2 in tsA-201 cells expressing Ca_V_2.2 (black squares, 100.0±10.6%, *n*=7) and Ca_V_2.2+FMRP (open red squares, 86.5±5.3%, *n*=7, *P*=0.27). The intensity of the signal was expressed relative to that of the GFP band in each experiment. Black bars represent the means±s.e.m.; one-way ANOVA. (**c**) Cell surface-biotinylation assay of tsA-201 cells expressing Ca_V_2.2 (left) or Ca_V_2.2+FMRP (right), plus Ca_V_β1b/Ca_V_α_2_δ-1. Biotinylated samples (Surface, top) and whole-cell lysates (Total, bottom) were immunoblotted with Abs against Ca_V_2.2 and Akt (as a control for lack of biotinylation of cytoplasmic proteins). (**d**) Cell surface-biotinylation assay of tsA-201 cells, expressing Ca_V_2.2 or Ca_V_2.2+FMRP, plus Ca_V_β1b and Ca_V_α_2_δ-1, treated with the proteasome inhibitor MG132 (5 μM) for 15 h. Biotinylated samples (Surface, top) and whole-cell lysates (Total, bottom) were immunoblotted with Ca_V_2.2 and Akt Abs. (**e**) Confocal images of non-permeabilized tsA-201 cells expressing HA-Ca_V_2.2+Ca_V_β1b+Ca_V_α_2_δ-1 without (left) or with FMRP (right), immunostained for HA (white). Scale bar, 20 μm applies to both images. (**f**) Confocal images of non-permeabilized tsA-201 cells expressing HA-Ca_V_2.2+Ca_V_β1b+Ca_V_α_2_δ-1 without (left) or with FMRP (right), treated with MG132, as in (**d**), immunostained for HA (white). Scale bar, 20 μm applies to both images. (**g**) Surface expression of Ca_V_2.2 in tsA-201 cells expressing HA-Ca_V_2.2 (filled bars) or HA-Ca_V_2.2+FMRP (open bars), plus Ca_V_β1b/Ca_V_α_2_δ-1. Left: The surface-biotinylated Ca_V_2.2 band was corrected for the intensity of the total Ca_V_2.2 in each experiment (Ca_V_2.2: 100.0±3.2%, *n*=16; Ca_V_2.2+FMRP: 71.3±7.8%, *n*=17, *P*=0.001). Right: HA surface immunoreactivity was normalized to the Ca_V_2.2 condition following correction for the background noise (Ca_V_2.2: 100.0±3.8%, *n*=200 cells; Ca_V_2.2+FMRP: 56.1±3.3%, *n*=198 cells, *P*<0.00001). Means±s.e.m., **P<0.01; ***P<0.001; one-way ANOVA. (**h**) Surface expression of Ca_V_2.2 in tsA-201 cells expressing Ca_V_2.2 (filled bars) or Ca_V_2.2+FMRP (red open bars), following treatment with MG132. Left: Surface-biotinylated Ca_V_2.2 (Ca_V_2.2: 100.0±4.2%, *n*=7; Ca_V_2.2+FMRP: 92.9±5.6%, *n*=7, *P*=0.33). Right: HA immunoreactivity (Ca_V_2.2: 100.0±4.3%, *n*=174 cells; Ca_V_2.2+FMRP: 102.4±5.6%, *n*=142 cells, *P*=0.72). Means±s.e.m.; one-way ANOVA.

To test for FMRP regulation of Ca_V_2.2 protein expression, we compared Ca_V_2.2 protein level in tsA-201 cells transfected with or without FMRP (Fig. 4a). We found that FMRP does not affect total Ca_V_2.2 protein level, or expression of the auxiliary subunits (Fig. 4b and Supplementary Fig. 2).

Therefore, we assessed plasma membrane expression of Ca_V_2.2 using a cell surface-biotinylation assay, and observed that biotinylated Ca_V_2.2 was significantly reduced in the presence of FMRP, by 28.7% (Fig. 4c–g). Ca_V_2.2 plasma membrane expression was further investigated by immunocytochemistry using an extracellular HA-tagged Ca_V_2.2 channel construct (Fig. 4e). We found that HA-Ca_V_2.2 immunoreactivity at the cell surface of non-permeabilized cells was reduced by 43.9% when Ca_V_2.2 was co-expressed with FMRP (Fig. 4g). The reduction of Ca_V_2.2 cell surface expression determined by both methods is in agreement with the reduction of current density that we observed (Fig. 2).

Ca_V_ channels are subject to degradation by the proteasome^20^,^21^. To test whether FMRP influences the targeting of cell surface-expressed Ca_V_2.2 to the proteasome, we examined the effect of the proteasome inhibitor MG132 on Ca_V_2.2 protein levels in the absence or presence of FMRP (Fig. 4d–f). We first confirmed that MG132 treatment was effective in blocking the degradation of Ca_V_2.2 expressed in tsA-201 cells (total Ca_V_2.2 level is increased by 56% in cells treated with MG132 compared with control, Supplementary Fig. 3). Then, using both the biotinylation assay and immunocytochemistry, we found that MG132 treatment abolished the reduction of Ca_V_2.2 surface expression induced by FMRP (Fig. 4h). Together, our results indicate that FMRP modulates Ca_V_2.2 expression at the plasma membrane by targeting the channels for proteasomal degradation.

### FMRP interacts with Ca_V_2.2 channels

**Figure 5:**
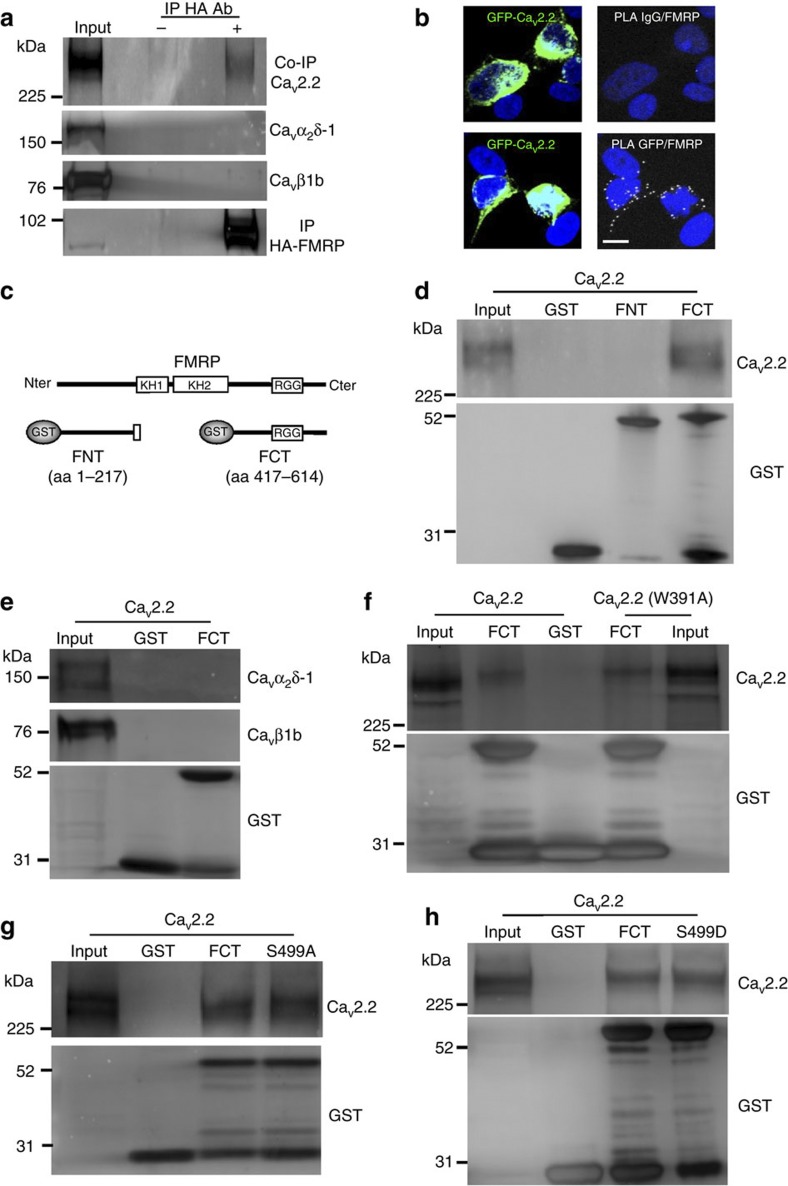
FMRP interacts with Ca_V_2.2 channels. (**a**) Ca_V_2.2 (top) co-immunoprecipitates with HA-tagged FMRP (bottom) in co-transfected tsA-201 cells, but not in control (Ab omitted). Ca_V_β1b and Ca_V_α_2_δ-1 were also transfected but neither were co-immunoprecipitated with HA-FMRP. Immunoblots were performed using Ca_V_2.2, Ca_V_α_2_δ-1, Ca_V_β1b and HA Ab. Representative of three experiments. (**b**) Confocal images of *in situ* PLA showing interaction between transfected GFP-tagged Ca_V_2.2 and HA-FMRP in tsA-201 cells. Ca_V_β1b and Ca_V_α_2_δ-1 were also transfected. PLA was performed using mouse FMRP Ab (7G1-1) and either rabbit IgG (top row) or rabbit anti-GFP Ab (bottom row). PLA signals (white, right panel) are detected only in cells positive for GFP-Ca_V_2.2 (green, left panel) when both GFP and FMRP Abs were used. 4',6-diamidino-2-phenylindole (blue) labels cell nuclei. Scale bar, 10 μm. Representative of three experiments. (**c**) Schematic depiction of FMRP and GST-fusion fragments used for pull-down assay. Nter, N-terminus; Cter, C-terminus; KH1 and KH2, K-homology domains 1 and 2; RGG, arginine-glycine-glycine box; FNT, GST-FMRP N-terminus; FCT, GST-FMRP C-terminus; aa, amino acid. (**d**) Western blots of pull-down assays show FCT, but not GST alone or FNT, binds Ca_V_2.2 expressed in tsA-201 cells plus Ca_V_β1b/Ca_V_α_2_δ-1. Input represents 5% of protein input included in the assay. Western blots (lower panel) show amount of GST-tagged protein used in the assay. Representative of three experiments. (**e**) Western blots of pull-down assays show neither Ca_V_α_2_δ-1 nor Ca_V_β1b expressed in tsA-201 cells (together with Ca_V_2.2) were pulled down with FCT. Input represents 5% of protein input included in the assay. Immunoblots were performed using Ca_V_α_2_δ-1 and Ca_V_β1b Ab. Representative of three experiments. (**f**) Ca_V_2.2 and mutant Ca_V_2.2 (W391A) expressed in tsA-201 cells (with Ca_V_α_2_δ-1/Ca_V_β1b) were both pulled down with FCT. Input represents 5% of protein input included in the assay. Representative of three experiments. (**g**,**h**) Western blots of pull-down assays show phosphorylation state of FMRP serine 499 does not modify interaction with Ca_V_2.2. (**g**) S499A, dephosphomimetic-FCT; (**h**) S499D, phosphomimetic-FCT. Input represents 5% of protein input included in the assay. Representative of three experiments. Full-size blots can be found in Supplementary Fig. 8.

To determine whether FMRP interacts with Ca_V_2.2, we first used immunoprecipitation on whole-cell lysate from tsA-201 cells expressing Ca_V_2.2 and HA-FMRP. We found that an HA Ab co-immunoprecipitates Ca_V_2.2 together with HA-FMRP (Fig. 5a). The interaction is likely to be directly with Ca_V_2.2 because HA-FMRP does not co-immunoprecipitate the co-expressed auxiliary Ca_V_β1b or Ca_V_α_2_δ-1 subunits (Fig. 5a), also pointing to the possibility that it might interact preferentially with channels that are not in functional complexes with their auxiliary subunits. The interaction between Ca_V_2.2 and FMRP was confirmed with an *in situ* proximity ligation assay (PLA). Using GFP Ab to detect GFP-Ca_V_2.2 and FMRP Ab to detect HA-FMRP, we found PLA-positive signals only in cells expressing GFP-Ca_V_2.2 (Fig. 5b); notably, the signal was present within the cytoplasm, as well as at the plasma membrane.

To identify which domain of FMRP is involved in interaction with Ca_V_2.2, we performed pull-down experiments using glutathione *S*-transferase (GST) fusion proteins with FMRP N-terminal or C-terminal domains (Fig. 5c). We applied whole-cell lysate from tsA-201 cells expressing Ca_V_2.2/Ca_V_β1b/Ca_V_α_2_δ-1 to each purified GST-fusion protein, and found that only the C-terminal domain of FMRP pulled down Ca_V_2.2 (Fig. 5d). We further showed that the C-terminal domain of FMRP is necessary for its functional effect because FMRP with a deletion of its C-terminal domain is unable to reduce Ca_V_2.2 current density (Supplementary Fig. 4). The interaction is specific for Ca_V_2.2 because neither Ca_V_β1b nor Ca_V_α_2_δ-1 was pulled down (Fig. 5e). We also found that Ca_V_2.2 containing a mutation (W391A) that prevents binding of Ca_V_β subunits to its I-II linker^22^ retains its ability to interact with the FMRP C-terminus, further suggesting that the interaction between FMRP and Ca_V_2.2 is independent of Ca_V_β (Fig. 5f). Finally, the interaction of FMRP with Ca_V_ channels appears to be specific for the Ca_V_2 family since Ca_V_2.1 but not Ca_V_1.2 channels binds to the FMRP C-terminus (Supplementary Fig. 5).

FMRP is phosphorylated primarily on the conserved S499 in its C-terminal domain^1^. To test whether this key serine is involved in the interaction with Ca_V_2.2, we mutated S499 into either an alanine (S499A, dephosphomimetic)^23^ or an aspartic acid (S499D, phosphomimetic)^5^. We performed GST pull-down experiments and found that neither mutation prevented the binding of FMRP C-terminus to Ca_V_2.2 (Fig. 5g,h).

### FMRP interacts with Ca_V_2.2 *in vivo*

**Figure 6:**
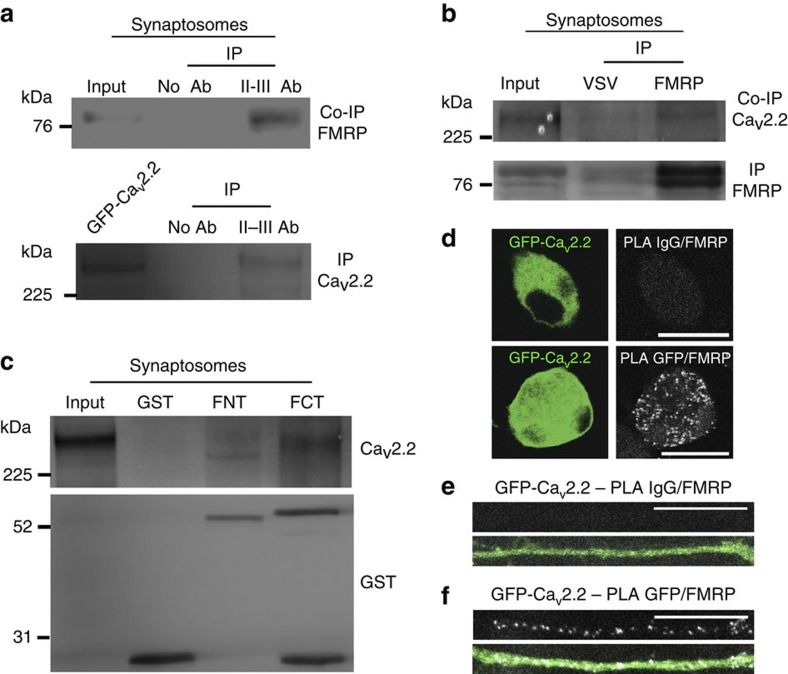
FMRP interacts with endogenous Ca_V_2.2 in neurons. (**a**) FMRP co-immunoprecipitates with endogenous Ca_V_2.2 from synaptosome extracts, but not in the control in which Ab was omitted (top). The ability of the rabbit polyclonal Ca_V_2.2 II–III loop Ab to immunoprecipitate Ca_V_2.2 was confirmed using whole-cell lysate from tsA-201 cells transfected with GFP-Ca_V_2.2 (bottom). A mouse monoclonal GFP Ab was used to detect GFP-Ca_V_2.2 expression. (**b**) Endogenous Ca_V_2.2 (top) co-immunoprecipitates with endogenous FMRP (bottom) from synaptosome extracts, but not in the control in which an unrelated Ab was used (VSV-g Ab). Immunoblots were performed using Ca_V_2.2 (top) and FMRP (bottom) Abs. (**c**) Western blots of pull-down assays revealed that FMRP C-terminus (FCT), but not GST alone or FMRP N-terminus (FNT), bound Ca_V_2.2 from synaptosome extracts (top). Input represents 5% of protein input included in the assay. Western blot (bottom) shows the amount of GST-fusion protein used in the assay. Full-size blots can be found in Supplementary Fig. 8. (**d**–**f**) Confocal images showing the interaction between transfected GFP-tagged Ca_V_2.2 and endogenous FMRP in DRG neurons. Ca_V_β1b and Ca_V_α_2_δ-1 were also transfected. *In situ* PLA was performed using mouse FMRP Ab (7G1-1) and either rabbit IgG (**d**, top row; **e**) or rabbit anti-GFP Ab (**d**, bottom row; **f**). PLA signals (white) are detected in soma (**d**) and process (**e**,**f**) of DRG positive for GFP-Ca_V_2.2 (green) when both GFP and FMRP Abs were used. Scale bar, 20 μm. Representative of three experiments.

We then examined whether the interaction between FMRP and Ca_V_2.2 occurs in neurons. We first showed that FMRP interacts with Ca_V_2.2 endogenously, by immunoprecipitation of either Ca_V_2.2 or FMRP from brain synaptosomes. We found that a Ca_V_2.2 Ab co-immunoprecipitates FMRP (Fig. 6a), and reciprocally we showed that an FMRP Ab co-immunoprecipitates Ca_V_2.2 (Fig. 6b). We also confirmed that the FMRP C-terminal domain interacts with synaptosomal Ca_V_2.2 (Fig. 6c). FMRP is expressed in DRG neurons throughout the soma and processes^24^ (Supplementary Fig. 6). To determine whether the interaction between FMRP and Ca_V_2.2 occurs in neurons, we transiently transfected DRG neurons with GFP-Ca_V_2.2 and performed *in situ* PLA using anti-GFP Ab and anti-FMRP Ab. We found PLA-positive signals in somata and processes of DRG neurons expressing GFP-Ca_V_2.2, showing that endogenous FMRP also forms protein complexes with Ca_V_2.2 both in DRG neuron cell bodies and in their neurites (Fig. 6d–f).

### FMRP interacts with Ca_V_2.2 synaptic targeting domains

**Figure 7:**
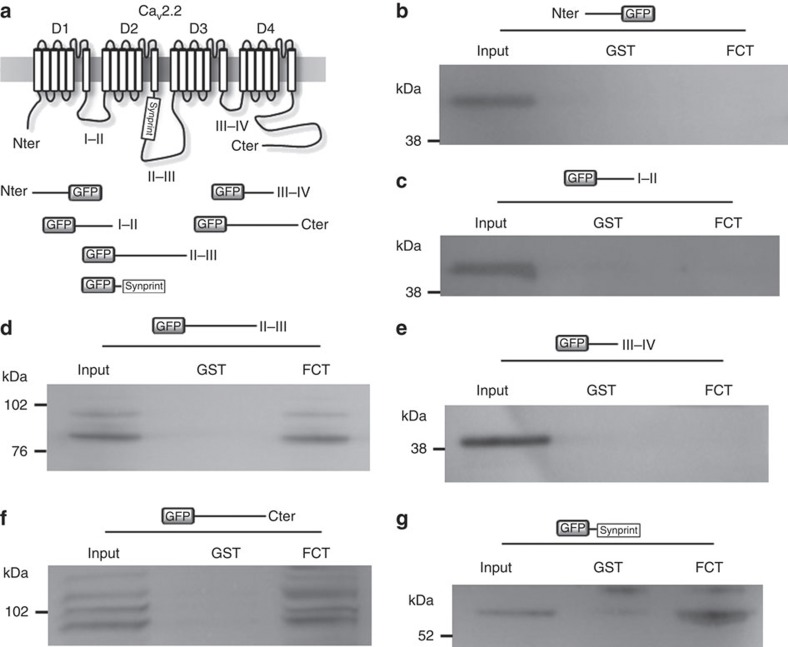
Intracellular domains of Ca_V_2.2 determining the interaction with FMRP. (**a**) Schematic depiction of Ca_V_2.2, together with GFP-tagged Ca_V_2.2 intracellular fragments used for the pull-down assay. D1, D2, D3 and D4: Domain 1 to domain 4; Nter, intracellular N-terminus; I–II, II–III, III–IV, cytoplasmic linkers; Synprint, amino acids 711–966 from the II–III linker; Cter, intracellular C-terminus. (**b**–**g**) Western blots of pull-down assays show that GST-FMRP C-terminus (FCT), expressed in yeast, bound to GFP-tagged Ca_V_2.2 II–III linker (**d**), Ca_V_2.2 C-terminus (**f**) and Ca_V_2.2 Synprint (**g**) expressed in tsA-201 cells. Input represents 5% of protein input included in the assay. Representative of 3 experiments in each case. Full-size blots can be found in Supplementary Fig. 8.

We then focused on identifying the domains of Ca_V_2.2 involved in the interaction with FMRP. We generated a series of GFP-tagged constructs corresponding to intracellular regions of Ca_V_2.2 (Fig. 7a). We expressed each construct individually in tsA-201 cells and applied the whole-cell lysate to immobilized GST-FMRP C-terminus (Fig. 7b–g). We found that the II–III linker (Fig. 7d) and the C-terminus of Ca_V_2.2 (Fig. 7f) were both pulled down by FMRP C-terminus, but the other intracellular regions were not (Fig. 7b,c,e). Interestingly, these two intracellular regions of Ca_V_2.2 are both involved in the interaction with presynaptic proteins^25^. The II–III linker contains a synaptic protein interaction (synprint)^25^ site and we found that this synprint motif on its own retains the ability to bind to FMRP C-terminus (Fig. 7g).

### FMRP controls synaptic release via N-type calcium channels

**Figure 8:**
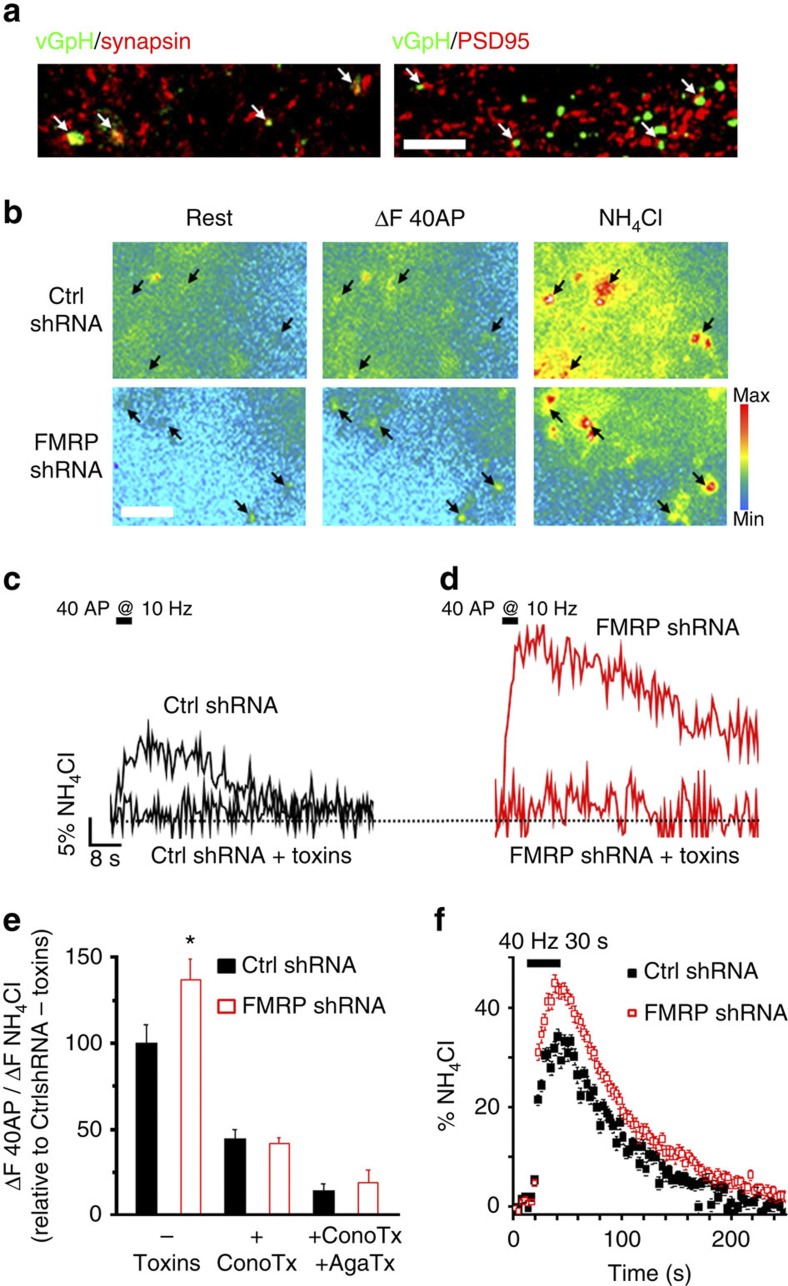
FMRP knockdown enhances synaptic vesicle exocytosis in presynaptic terminals of DRG neurons via Ca_V_2.2 channels. (**a**) Presynaptic terminals of DRG neurons expressing vGpH (vGpH). Images show vGpH fluorescence (green) colocalized with endogenous synapsin 1 and 2 (left panel, red) and apposed to endogenous PSD-95 (right panel, red). Synapses are indicated by the white arrows. Scale bars, 5 μm. (**b**) Fluorescence changes (ΔF) of vGpH in presynaptic terminals of DRG neurons transfected with Ctrl shRNA (top panels) or FMRP shRNA (bottom panels) in response to electrical stimulation. Left panels: at rest; middle panels: after 40 action potentials (AP) at 10 Hz; right panels: after a brief application of NH_4_Cl. Responsive terminals are indicated by the black arrows. Pseudocolor scale is shown to the right (min, max:minimum and maximum fluorescence intensity). Scale bar, 10 μm. (**c**,**d**) vGpH response to 40 AP at 10 Hz from presynaptic terminals of DRG neurons transfected with Ctrl shRNA (**c**) or FMRP shRNA (**d**) before and after treatment with toxins (10 min with ω-conotoxin GVIA (1 μM) and ω-agatoxin IVA (300 nM)). Fluorescence intensities were normalized to the peak of a brief application of NH_4_Cl. (**e**) Normalized vGpH responses to 40 AP at 10 Hz from presynaptic terminals of DRG neurons transfected with Ctrl shRNA (black-filled bar, 100±10.6%, *n*=38) or FMRP shRNA (red open bar, 137.0±12.6%, *n*=25, *P*=0.027). ω-conotoxin GVIA (ConoTx, 1 μM) reduces Ctrl shRNA and FMRP shRNA responses to a similar level (44.7±4.9%, *n*=15 and 41.6±3.3%, *n*=24, respectively). ω-conotoxin GVIA (1 μM) and ω-agatoxin IVA (AgaTx, 300 nM) application reduces further the responses: Ctrl shRNA=17.3±3.2%, *n*=38, and FMRP shRNA=18.1±5.0%, *n*=27. A dot plot graph for the data is presented in Supplementary Fig. 9. Means±s.e.m., *P<0.05; one-way ANOVA. (**f**) Average vGpH response to a 40 Hz stimulation for 30 s from presynaptic terminals of DRG neurons transfected with Ctrl shRNA (black-filled squares) or FMRP shRNA (open red squares). The decay of the signal after stimulation is well fitted by a monoexponential: *τ*=20.3±2.3 s for Ctrl shRNA (*n*=111) and *τ*=20.9±0.9 s for FMRP shRNA (*n*=77). Means±s.e.m.

Ca_V_2 channels have a major presynaptic role in regulation of transmitter release^12^,^13^, and Ca_V_2.2 is particularly important in the peripheral nervous system. In order to determine the physiological impact of the interaction between FMRP and Ca_V_2.2 channels, we assessed the effect of loss of FMRP on synaptic vesicle release. We used the vGlut1-pHluorin (vGpH) optical reporter to measure synaptic vesicle recycling in DRG neurons co-cultured with DH neurons^26^. E18 DRG neurons were co-transfected with mCherry, vGpH and either Ctrl shRNA or FMRP shRNA and then plated with untransfected DH neurons. DRG neurons were imaged after 9–11 days in culture. vGpH colocalized in varicosities with presynaptic marker synapsin and was in apposition to postsynaptic marker PSD-95 (Fig. 8a). We monitored the increase in fluorescence of vGpH in response to 40 action potentials at 10 Hz (Fig. 8b). Signals from each bouton were normalized to the fluorescence value obtained by rapid alkalinization of the entire labelled vesicle pool using NH_4_Cl (Fig. 8b). We found that responses to 40 action potentials at 10 Hz were increased by 37% in synapses from DRG neurons in which FMRP was knocked down compared with control (Fig. 8c–e). During stimulation, the change of vGpH signal reflects the difference between exocytosis and ongoing vesicle endocytosis and reacidification^27^. After stimulation, the signal decays due to endocytosis of vGpH and vesicle reacidification. We then examined synaptic vesicle recycling by stimulating neurons at 40 Hz for 30 s and monitoring the time course of fluorescence decay. We found that the rate of decay was not different in boutons of DRG neurons lacking FMRP compared with controls (Fig. 8f). We can then conclude that the increase of vesicle release during stimulation of synaptic terminals of which FMRP was knocked down is due to an increase in exocytosis. Interestingly, we recorded a similar increase in vesicle release in synapses from the hippocampal neurons expressing FMRP shRNA (37.5% increase compared with Ctrl shRNA, Supplementary Fig. 7), suggesting that FMRP is also involved in vesicular release in central nervous system synapses.

Using specific blockers for Ca_V_2.2 and Ca_V_2.1 channels (ω-conotoxin GVIA and ω-agatoxin IVA, respectively), we showed that in control synapses 55.3% of the exocytosis was inhibited by ω-conotoxin GVIA and that 82.7% was blocked by ω-conotoxin GVIA+ω-agatoxin IVA co-application (Fig. 8c–e). Remarkably, at synapses of DRG neurons in which FMRP was knocked down, ω-conotoxin GVIA reduced exocytosis to a level comparable with the control (inhibition of 58.4%, Fig. 8e) and therefore abolished the increase induced by the lack of FMRP. Finally, ω-conotoxin GVIA together with ω-agatoxin IVA reduced exocytosis by 82% in DRG neurons in which FMRP was knocked down (Fig. 8e). Together, these data demonstrate that FMRP regulates vesicle exocytosis via Ca_V_2.2 channels in DRG neurons.

## Discussion

FXS, the most common heritable form of mental retardation, is characterized by synaptic dysfunction and is caused by the loss of FMRP^1^,^2^. Regulation of Ca_V_ expression at presynaptic terminals is a critical factor in the control of synaptic transmission. In this study, we show that FMRP exerts a tonic inhibition of somatic Ca_V_ current density and Ca_V_2.2 surface expression at presynaptic terminals of DRG neurons. We also show that FMRP reduces Ca_V_2.2-generated currents by decreasing the expression of the channels at the plasma membrane, and we provide evidence that this mechanism depends on proteasomal degradation. We demonstrate that the interaction between FMRP and Ca_V_2.2 is direct, since we find that the C-terminal domain of FMRP binds to the Ca_V_2.2 II–III linker and its C-terminal domain. Finally, we show that FMRP controls synaptic vesicle exocytosis via Ca_V_2.2 channels. Our study demonstrates a hitherto unknown effect of FMRP on Ca_V_2.2 expression and indicates a major presynaptic function for FMRP.

In this study, we show that FMRP affects Ca_V_2.2 cell surface expression at the presynaptic element of DRG neurons and modulates synaptic transmission by this means. Consistent with this hypothesis, studies in animal models of FXS show an increase in transmitter release at the *Drosophila* neuromuscular junction^7^,^8^ and an increase of synaptic vesicle recycling in mouse hippocampal neurons^10^. However, the lack of FMRP has also been associated with decrease of synaptic transmission^7^,^28^,^29^, and FMRP appears to regulate multiple synaptic parameters depending on the developmental stage and the area of the nervous system.

Ca_V_2.1 and Ca_V_2.2 channels are the two major classes of Ca_V_ in presynaptic terminals, whereas Ca_V_1.2 channels are located mainly in postsynaptic elements^14^. We show that FMRP interacts with Ca_V_2.1 and Ca_V_2.2, but not with Ca_V_1.2 channels, suggesting that the interaction is specific for presynaptic calcium channels (Supplementary Fig. 5). We also show that FMRP interacts with the II–III linker synprint site and C-terminal region of Ca_V_2.2. Several presynaptic proteins have been found to interact with Ca_V_2.2 channels, including SNARE proteins syntaxin 1A and SNAP-25 (refs 25, 30) and active-zone proteins including Rab-interacting molecule (RIM)^31^. The interaction between SNARE proteins and Ca_V_2.2 synprint site has been found to modulate the targeting of the Ca_V_2.2 channel to the nerve terminals of both peripheral sympathetic ganglion neurons^32^ and central hippocampal neurons^33^ and also to inhibit calcium currents^34^. Moreover, Ca_V_2.2 channels, via a postsynaptic density-95/discs large/zona occludens-1 (PDZ) domain binding motif and a proline-rich region located in the Ca_V_2.2 C-terminal domain, can directly interact with Mint, CASK, RIM1 and RIM-BP2 to target the channels to presynaptic active zones^35^,^36^. G protein-coupled receptor have also been shown to associate with Cav2.2 via the II–III linker and the C-terminal domain^37^ and this can alter the expression of the Cav2.2 channels at the plasma membrane^38^,^39^. These interactions provide an effective association between Ca^2+^ entry and the vesicle release sites that ensures the rapid triggering of neurotransmitter release when an action potential invades the nerve terminal. Precisely how the interaction with FMRP affects Ca_V_2.2 synaptic localization is still unclear and further investigation is required to elucidate this process.

Several mechanisms can control Ca_V_ expression at the plasma membrane; among them interactions with the auxiliary Ca_V_α_2_δ-1 subunits markedly affect their functional expression^11^. We show that FMRP does not affect Ca_V_α_2_δ-1 expression and does not interact with Ca_V_α_2_δ-1, making it unlikely that FMRP affects Ca_V_2.2 surface expression via a Ca_V_α_2_δ subunit-dependent mechanism, despite the role of Ca_V_α_2_δ proteins in presynaptic calcium channel targeting^26^. Trafficking and surface expression of Ca_V_ also depends on the interaction with auxiliary Ca_V_β subunits^11^. Recent studies have shown that the interaction of Ca_V_1 and Ca_V_2 channels with Ca_V_β subunits protects these channels against proteasomal degradation^20^,^21^. Interestingly, we find here that the effect of FMRP on Ca_V_2.2 expression is antagonized by a proteasome inhibitor, and we found that the interaction between FMRP and Cav2.2 does not co-immunoprecipitate Ca_V_β subunits, suggesting that it occurs with channels that have lost, or subsequently lose, the protective interaction with Ca_V_β subunits^11^. Nevertheless, further work is needed to unravel the molecular mechanism by which FMRP targets Ca_V_2.2 for degradation. For example, does FMRP also interact with an element of the proteasome and thus target Ca_V_2.2 to this pathway, in which case FMRP would play the role of a molecular adaptor?

Our findings reveal that FMRP interacts directly with the Ca_V_2.2 channel protein, both *in vitro* and in cultured DRG neurons. A recent study has reported that FMRP can directly interact and modulate the gating of the sodium-activated potassium channel Slack-B^15^. Whereas in this latter report, the FMRP N-terminal domain is shown to be involved in the interaction with Slack-B, we find here that the FMRP C-terminal domain interacts with Ca_V_2.2. The FMRP N-terminal domain is a well-described platform for protein–protein interactions^40^ but only two reports have so far described protein–protein interactions with FMRP C-terminal domain: the motor protein kinesin light chain involved in RNA granule and axonal transport^3^ and the scaffolding protein Ran-binding protein in the microtubule-organizing centre (RanBPM), which is associated with synapse formation in neurons^41^,^42^. The FMRP C-terminal domain is a non-conserved region in the related proteins FXR1P and FXR2P^1^. Altogether, these data reinforce the hypothesis that the FMRP C-terminal domain contributes to determining the specificity of FMRP function^41^.

More recently, FMRP has been shown to interact with large-conductance calcium-activated potassium (BK) channel via their auxiliary β4 subunit and to regulate neurotransmitter release at hippocampal CA3-CA1 synapses by modulating action potential duration in the soma of CA3 neurons^16^. This latter study also described that the intracellular perfusion of FMRP Ab induces an increase of calcium transient in presynaptic boutons of CA3 neurons, although the mechanism behind this increase of calcium transient was not examined. BK channels are modulators of action potential duration in the soma of CA3 neurons, but their involvement in presynaptic vesicle exocytosis is still a matter of debate^43^. Further investigation is needed to identify the mechanism by which FMRP modulates the calcium transient in the CA3-CA1 hippocampal synapses but our results support the view of an involvement of Ca_V_2 channels.

Taken together, our data demonstrate a direct and tonic role for FMRP in regulating presynaptic N-type calcium channel expression and function in DRG neurons. Presynaptic Ca_V_2.2 channels are critical for neurotransmission, both in central neurons and in the autonomic and sensory nervous system, where they are involved in nociception and short-term synaptic plasticity^14^,^44^. Therefore, dysregulation of Ca_V_2.2 expression could account for aspects of the cognitive impairment and altered pain sensitivity observed both in patients and in a mouse model of FXS^24^,^45^,^46^. Consistent with this hypothesis, a clinical trial using a γ-aminobutyric acid type B (GABA_B_) agonist (R-baclofen), an inhibitory modulator of N-type calcium channels^47^, found some improvement of social function and behaviour in FXS patients^48^. Our study opens the possibility that small molecule inhibitors of Ca_V_2.2, currently being developed for a number of chronic pain conditions^49^, could be of benefit in FXS.

## Methods

### cDNA constructs

The following cDNAs were used: Ca_V_2.2 (D14157), Ca_V_2.1 (M64373), Ca_V_1.2 (M67515), Ca_V_β1b (X61394; from Dr T. P. Snutch,), Ca_V_α_2_δ-1 (Genbank accession number AF_286488), mut-3b GFP(M62653, except S72A and S65G, Dr T. E. Hughes), all subcloned in pMT2 for expression in tsA-201 cells and subcloned in pcDNA3.0 or pRK5 vectors for expression in neurons. peGFP-FMRP (NBCI nucleotide accession code NM_008031.2) was a gift from Dr G. J. Bassell; Vamp-mCherry and vGlut-pHluorin was a gift from Dr T.A. Ryan. The HA-tagged Ca_V_2.2 construct was generated with an HA tag in an extracellular linker, similar to that previously described^20^. Truncated and GFP-tagged Ca_V_2.2 constructs (Nter-GFP, I-II-GFP, GFP-II–III, GFP-synprint, GFP-III–IV and GFP-Cter) and HA-FMRP were generated using standard molecular biological techniques and confirmed by DNA sequencing. All constructs were cloned into the pcDNA3.0 vector for expression in mammalian cells. shRNA plasmids were generated as previously described^50^ using the following mRNA target sequence for FMRP: 5′-GTGATGAAGTTGAGGTTTA-3′ (ref. 51).

### Cell culture and transfection

tsA-201 cells (96121229, ECACC) were cultured in Dulbecco's modified Eagle's medium with 10% fetal bovine serum (FBS), penicillin and streptomycin, and 2% GlutaMAX (Invitrogen). tsA-201 cells were transfected using FuGene6 reagent (Roche and Promega), following the manufacturer’s instructions. P10 DRG neurons were prepared from male Sprague Dawley rats and transfected using Amaxa Nucleofector (Lonza) according to the manufacturer’s protocol. E18 and P2 rat DRG/DH neuron co-cultures were prepared from isolated DRGs and spines. Neurons were dissociated by trypsinization (0.25%) followed by trituration. Neurons were plated on glass coverslips coated with poly-L-lysine and laminin and cultured in Neurobasal medium containing B27 supplement, penicillin and streptomycin, NGF (100 ng ml^−1^), and 2% GlutaMAX (Invitrogen). One-half of the growth medium was replaced every 3 days. DRG neurons were transfected before plating using Amaxa Nucleofactor (Lonza) according to the manufacturer’s instructions or microinjected (Eppendorf microinjection system) after 7 days in culture.

### Western blot analysis

At 48 h after transfection, cells were rinsed twice with phosphate-buffered saline (PBS) and then harvested in PBS containing protease inhibitors (Complete tablet from Roche). The cells were lysed in PBS, 1% Igepal and protease inhibitors for 30 min on ice. The detergent lysates were then clarified by centrifugation (14,000 *g*, 30 min, 4 °C). Proteins were separated by SDS–PAGE on 3–8% Tris-acetate or 4–12% Bis-Tris gels and then transferred to polyvinylidene fluoride membranes. After blocking in Tris-buffered saline buffer (10 mM Tris, pH 7.4, 500 mM NaCl. 0.5% Igepal, 10% goat serum and 3% BSA), the membranes were incubated with primary antibody overnight. The protein–Ab complexes were then labelled with a horseradish peroxidase-conjugated secondary Ab (1:3,000 Sigma-Aldrich) for 1 h at room temperature and detected using the enhanced ECL Plus reagent (GE Healthcare) visualized with a Typhoon 9410 scanner (GE Healthcare). Quantification of immunoblot bands was performed with ImageQuant software (GE Healthcare). The following Abs were used: rabbit anti-Ca_V_2.2 (1:500)^52^, rabbit anti-Ca_V_β1b (1:500) and mouse anti-Ca_V_α_2_δ-1 (1:3,000, D219, Sigma).

### Synaptosomal fraction preparation

Synaptosomal fractions were prepared by differential centrifugation^53^. Whole rat brains were homogenized in: 0.32 M sucrose, 3 mM HEPES-Na, pH 7.4 containing protease inhibitors. The homogenate was centrifuged at 1,000 *g* for 10 min to produce a pellet (P1) and a supernatant (S1). The pellet P1 was resuspended in homogenization buffer and centrifuged at 1,000*g* for 10 min to produce a pellet (P1′) and a supernatant (S1′). S1 and S1′ were combined and centrifuged at 12,000 *g* for 15 min to produce a pellet P2 and a supernatant S2. P2 was resuspended in homogenization buffer and centrifuged 15 min at 13,000 g to produce the crude synaptosomal fraction P2′. P2′ was resuspended in 1% Igepal PBS, incubated for 30 min on ice and clarified by centrifugation 30 min at 12,000 *g*.

### Immunoprecipitation

Clarified cell lysates or synaptosomal fractions were cleared with 50 μg of protein A sepharose (GE Healthcare) for 1 h at 4 °C. Supernatants were incubated with 2 μg ml^−1^ of specific Ab overnight at 4 °C with constant agitation. A further 20 μg of protein A sepharose was added and incubated for 1 h at 4 °C. Beads were washed three times with PBS containing 0.1% Igepal and incubated for 15 min at 55 °C with 100 mM dithiothreitol and 2X Laemmli sample buffer. Eluted proteins were then resolved by SDS–PAGE. The following Abs were used: rat anti-HA (Roche), MAB 7G1-1 anti-FMRP (Developmental Studies Hybridoma Bank, University of Iowa), MAB2160 anti-FMRP (Millipore) and rabbit anti-Ca_V_2.2.

### Cell surface biotinylation

At 18 h after transfection, cells were rinsed twice with PBS and then incubated with PBS containing 1 mg ml^−1^ Sulfo-NHS-SS-Biotin (Perbio) for 30 min at room temperature and then rinsed once with PBS and twice with PBS containing 200 mM glycine. Cells were then harvested and lysed in PBS, 1% Igepal and protease inhibitors for 30 min on ice. The detergent lysates were then clarified by centrifugation (14,000 × g, 30 min, 4 °C). Biotinylated proteins were precipitated by adding 100 μl of streptavidin-agarose beads (Perbio) and incubated overnight at 4 °C. The streptavidin-agarose beads were washed three times and incubated for 1 h at 37 °C with 100 mM dithiothreitol and 2x Laemmli sample buffer. Eluted proteins were then resolved by SDS–PAGE. The following Abs were used: rabbit anti-Ca_V_2.2 (1:500)^52^ and rabbit polyclonal anti-Akt (9272, 1:1,000, Cell Signalling Technology).

### GST pull-down assay

For pull-down assays, GST was subcloned into pYES2.1/V5-His TOPO TA (Invitrogen) by inserting PCR product using pGEX-2T as a template (GE Healthcare). GST-tagged constructs were generated by inserting PCR products of the mouse FMRP N-terminal (nucleotides 266–1003) and C-terminal (nucleotides 1514–2104) into *Eco*RI site of a pYES2.1/V5-His-GST. Yeasts (*Saccharomyces cerevisiae*) were transformed with individual expression vectors encoding the GST-fusion proteins and produced by standard methods. The yeast was lysed by vigorous shaking in PBS containing protease inhibitors (Complete tablet, Roche) and glass beads (Sigma) at 4 °C for 20 min. The lysates were then clarified by centrifugation (14,000 *g*, 5 min, 4 °C). GST-fusion proteins were immobilized on glutathione sepharose 4B beads (GE Healthcare) and incubated at 4 °C with lysate from tsA-201 cells transfected with full-length Ca_V_2.2 or GFP-tagged Ca_V_2.2 constructs or synaptosomal fraction preparations. Beads were washed four times with ice-cold 1% Triton-PBS containing protease inhibitors (Complete tablet, Roche) and incubated for 15 min at 55 °C with 100 mM dithiothreitol and 2X Laemmli sample buffer. Eluted proteins were then resolved by SDS–PAGE. The following Abs were used: rabbit polyclonal anti-Ca_V_2.2 (ref. 52) (1:500), mouse monoclonal anti-GST (sc-138, 1:3,000, Santa Cruz Biotechnology) and mouse monoclonal anti-GFP (632380, 1:3,000, Clontech).

### Immunocytochemistry

Cells were fixed with 4% paraformaldehyde/sucrose in PBS for 5 min and then permeabilized with 0.1% Triton X-100 in PBS for 10 min at room temperature. Cells were blocked with 10% FBS in PBS for at least 30 min at room temperature, and incubated with the primary Ab overnight with 3% FBS in PBS. Primary antibodies used were as follows: rabbit anti-βIII-tubulin (T2200, 1:1,000, Sigma), rabbit anti-synapsin 1, 2 (106002, 1:200, Synaptic systems), mouse anti-PSD-95 (ab2723, 1:200, Abcam). Samples were then washed and incubated with secondary conjugated Ab (1:500; anti-rabbit AF594, A11072; anti-rabbit AF633, A31573; anti-mouse AF594, A11005; anti-mouse AF633, A21050; all from Invitrogen and anti-mouse FITC, F2012, from Sigma) for 1 h at room temperature. After washing, samples were mounted in VectaShield (Vector Laboratories). Cells were examined on LSM 510 Meta or LSM 780 confocal microscopes (Zeiss).

### *In situ* PLA

Cells were fixed with 4% paraformaldehyde/sucrose in PBS for 5 min, incubated in 0.1 M Tris HCl for 5 min at room temperature and then permeabilized with 0.1% Triton X-100 in PBS for 10 min at room temperature. Cells were blocked with 10% FBS/0.1% Tween 20 in 4 × SSC for 30 min at 37 °C. Cells were incubated overnight with the primary antibody pair (1:500) from different species, directed against GFP (rabbit polyclonal anti-GFP, TP-401; Torrey Pines) and FMRP (mouse monoclonal MAB 7G1-1, Developmental Studies Hybridoma Bank, University of Iowa) and thereafter subjected to *in situ* PLA using Duolink Detection kit (Olink Bioscience) according to the manufacturer’s instruction. Briefly, after incubation with primary antibodies, the cells were incubated with secondary antibodies conjugated with oligonucleotides (mouse PLA probe MINUS and rabbit PLA probe PLUS, supplied in the PLA kit). Subsequently, connector oligonucleotides and ligase were added; the connector oligonucleotides hybridize to the two PLA probes and join to form a circular DNA strand when the PLA probes are in close proximity. After ligation, a polymerase is added and rolling-circle amplification is initiated using one of the PLA probes as a primer. The amplification product is detected through hybridization of fluorescently labelled oligonucleotides. Thus, individual pairs of proteins generate a spot that can be visualized using fluorescence microscopy. The theoretical maximum distance between the 2 target proteins is 30–40 nm to be able to create a PLA signal.

### Live cell imaging

Coverslips were mounted in a rapid-switching, laminar-flow perfusion and stimulation chamber (Warner Instruments) on the stage of an epifluorescence microscope (Axiovert 200 M, Zeiss). Live cell images were acquired with an Andor iXon+ (model DU-897U-CS0-BV) back-illuminated EMCCD camera using OptoMorph software (Cairn Research, UK). White and 470 nm LEDs served as light sources (Cairn Research, UK). Fluorescence excitation and collection was done through a 40 × 1.3 NA Fluar Zeiss objective using 450/50 nm excitation and 510/50 nm emission and 480 nm dichroic filters (for pHluorin) and a 572/35 nm excitation and low-pass 590 nm emission and 580 nm dichroic filters (for mCherry). Action potentials were evoked by passing 1 ms current pulses via platinum electrodes. Cells were perfused (0.5 ml min^−1^) in a saline solution at 30 °C containing (in mM) 119 NaCl, 2.5 KCl, 2 CaCl_2_, 2 MgCl_2_, 25 HEPES (buffered to pH 7.4), 30 glucose, 10 μM 6-cyano-7-nitroquinoxaline-2,3-dione (CNQX) and 50 μM D,L-2-amino-5-phosphonovaleric acid (AP5, Sigma). ω-conotoxin GVIA (1 μM) and ω-agatoxin IVA (300 nM) were applied for 10 min before stimulation (Alomone Labs). NH_4_Cl applications were done with 50 mM NH_4_Cl in substitution of 50 mM of NaCl (buffered to pH 7.4). Images were acquired at 2 Hz and analysed in ImageJ (http://www.rsb.info.nih.gov/ij) using a custom-written plugin (http://www.rsb.info.nih.gov/ij/plugins/time-series.html).

### Electrophysiology

Whole-cell patch-clamp recordings were performed on tsA-201 cells or DRG neurons at room temperature (21–25 °C). Single cells were voltage clamped using an Axopatch 200B patch-clamp amplifier (Axon instruments). Patch pipettes were filled with a solution containing the following (in mM): 140 Cs-aspartate, 5 EGTA, 2 MgCl_2_, 0.1 CaCl_2_, 2 K_2_ATP and 10 HEPES, titrated to pH 7.2 with CsOH. The external solution contained the following (in mM): 150 tetraethylammonium bromide, 3 KCl, 1 NaHCO_3_, 1 MgCl_2_, 10 HEPES, 4 glucose and 1 BaCl_2_, pH adjusted to 7.4 with Tris base. Recordings from DRG neurons were performed using 10 mM BaCl_2_. Current density–voltage relationships were fitted with a modified Boltzmann equation as follows: (1) *I*=*G*_max_ × (*V−V*_rev_)/(1+exp(−(*V−V*_50,act_)/*k*)), where *I* is the current density (in pA/pF), *G*_max_ is the maximum conductance (in nS/pF), *V*_rev_ is the reversal potential, *V*_50,act_ is the midpoint voltage for current activation and *k* is the slope factor. Steady-state inactivation and activation data were fitted with a single Boltzmann equation of the following form: (2) *I*/*I*_max_=(*A*_1_−*A*_2_)/[1+exp((*V*−*V*_50,inact_)/*k*/]+*A*_2_), where *I*_max_ is the maximal current, and *V*_50,inact_ is the half-maximal voltage for current inactivation. For the steady-state inactivation, *A*_1_ and *A*_2_ represent the proportion of inactivation and non-inactivating current, respectively. Inactivation kinetics of the currents were estimated by fitting the decaying part of the current traces with the following equation: (3) *I*(*t*)=*C*+*A* × exp(−(*t−t*_0_)/*τ*_inact_), where *t*_0_ is zero time, *C* the fraction of non-inactivating current, *A* the relative amplitude of the exponential, and *τ*_inact_ is the time constant.

### Non-stationary fluctuation analysis

Fluctuation analysis was carried out using the method described by Sigworth^54^. Ensembles of currents were generated by a series of identical voltage pulses delivered every 5 s. Currents were recorded using 10 mM BaCl_2_ and filtered at 2 kHz. The variance was calculated between successive sweeps and then averaged over all the pairs. Background variance at the holding potential was subtracted from the variance during the test pulse. Single-channel current was estimated by plotting the variance as a function of the mean current and fitting the data by (4) variance=*i* × *I*−*I*^2^/*N*, where *i* is the unitary current, *I* is the mean current and *N* is the number of functional channels^55^.

### Gating currents

ON-gating currents were measured during test pulses to positive potentials at which no ionic inward or outward currents were observed. Currents were filtered at 10 kHz. Patch pipettes were filled with a solution containing the following (in mM): 150 *N*-methyl-D-glucamine (NMDG), 10 EGTA, 1 MgCl_2_, 10 HEPES and 4 Mg-ATP (adjusted to pH 7.3 with methanesulfonic acid). The external solution contained the following (in mM): 135 choline chloride, 4 MgCl_2_, 10 Hepes and 1 CaCl_2_; pH adjusted to 7.2 with CsOH.

### *G*/*Q* analysis

*G*/*Q* analyses were performed as previously described^19^. *G*_max_ was determined for each cell as the slope of the peak current–voltage relationship (between +20 mV and +60 mV). *G*_max_ is related to single-channel open probability at maximal depolarization by the following equation: (5) *G*_max_=*P*_Omax_*ng*, where *P*_Omax_ is the single-channel open probability at maximal depolarization, *n* is the number of channels and *g* is the single-channel conductance. *Q*_max_ can be determined according to the following equation: (6) *Q*_max_=*nq*_max_, where *q*_max_ is the maximum gating charge moved per single channel. Plotting *G*_max_ as a function of *Q*_max_ defines a linear relationship with a slope (7) *G*_max_/*Q*_max_=*P*_Omax_(*g*/*q*_max_). Assuming that the single-channel conductance is not modified (Fig. 3c) and that the number of elementary charges moved are the same (voltage dependence of activation of their gating currents are indistinguishable, Fig. 3g) then the slope *G*_max_/*Q*_max_ is proportional to *P*_Omax_.

### Statistical analysis

Statistical analysis was conducted by one-way analysis of variance followed by Tukey’s multiple comparison tests. Statistical significance was set at *P*<0.05, and all error bars represent s.e.m.

## Additional information

**How to cite this article**: Ferron, L. *et al*. Fragile X mental retardation protein controls synaptic vesicle exocytosis by modulating N-type calcium channel density. *Nat. Commun.* 5:3628 doi: 10.1038/ncomms4628 (2014).

## Supplementary Material

Supplementary Information Supplementary Figures 1-9
