# Supplementary material for: Fragile X mental retardation protein controls synaptic vesicle exocytosis by modulating N-type calcium channel density
Source: Nat Commun. 2014 Apr 7;5:3628. doi: 10.1038/ncomms4628 (PMC3982139; doi:10.1038/ncomms4628)
Supplement: Supplementary Information — Supplementary Figures 1-9 [file ncomms4628-s1.pdf]

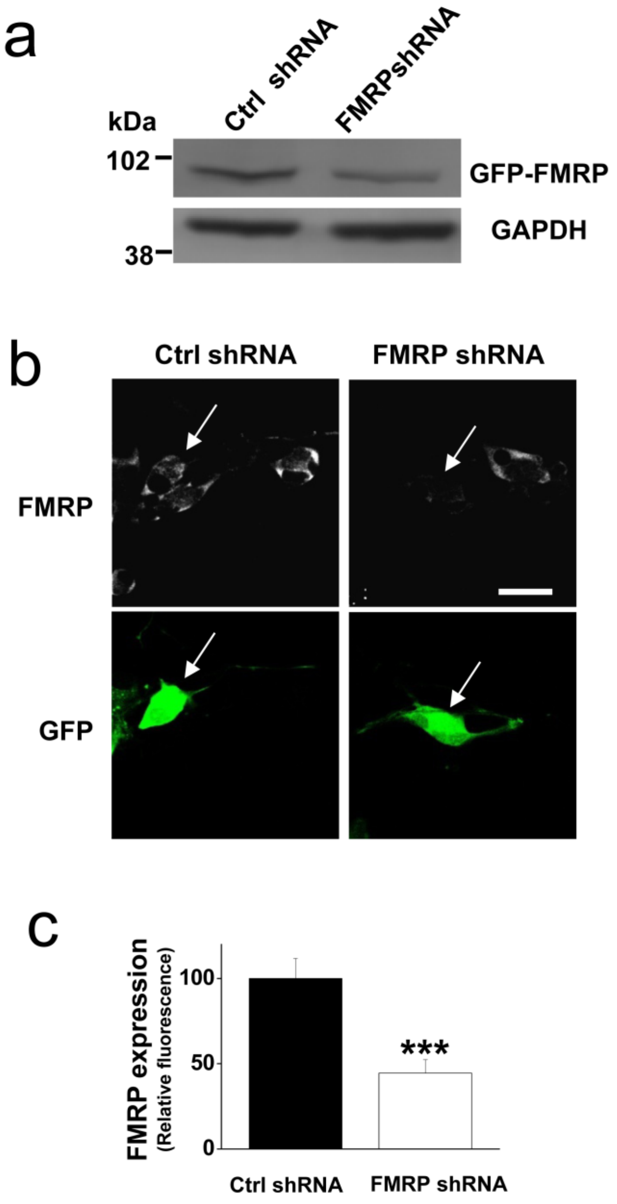

Supplementary Figure 1. Validation of FMRP knock-down in tsA-201 cells.

(a) Western blots of GFP-FMRP in tsA-201 cells 4 days after co-transfection with control (Ctrl) shRNA or FMRP shRNA. FMRP expression is reduced by 52% with FMRP shRNA (Ctrl shRNA=100±11%, n=5; FMRP shRNA=48±9%, n=5, *P*=0.0008). GAPDH was used as a loading control. Immunoblots were performed using GFP and GAPDH. (b) Confocal images showing reduced expression of FMRP in DRG neurons 4 days after transfection with FMRP shRNA (top right) but not control (Ctrl) shRNA (top left). FMRP immunostaining is pseudo-colored white. GFP (bottom row) shows transfected neurons. Scale bar, 20μm. (c) Bar chart showing normalized FMRP immunostaining density in DRG neurons 4 days after transfection with either Ctrl shRNA (filled bar, 100±12%, n=17) or FMRP shRNA (open bar, 44±8%, n=15, *P*=0.0006). Means±s.e.m., ****P*<0.001; one-way ANOVA.

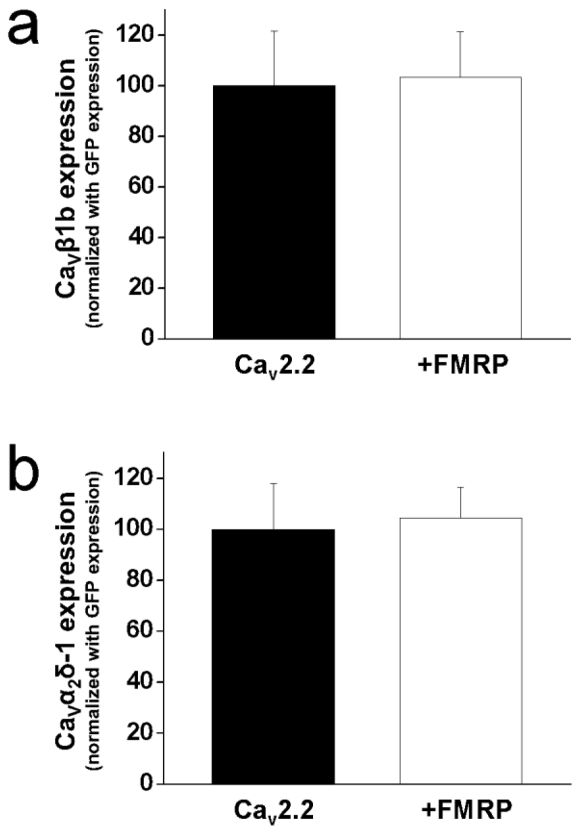

Supplementary Figure 2. FMRP has no effect on $Ca_V\beta1b$ and $Ca_V\alpha_2\delta$-1 auxiliary subunits expression.

(a) Normalized $Ca_V\beta1b$ protein levels in tsA-201 cells expressing $Ca_V2.2+Ca_V\beta1b+Ca_V\alpha_2\delta$-1 (100±22 %, n=8) or $Ca_V2.2+Ca_V\beta1b+Ca_V\alpha_2\delta$-1+HA-FMRP (103±18%, n=8, *P*=0.905). (b) Normalized $Ca_V\alpha_2\delta$-1 protein levels in tsA-201 cells expressing $Ca_V2.2+Ca_V\beta1b+Ca_V\alpha_2\delta$-1 (100±18%, n=8) or $Ca_V2.2+Ca_V\beta1b+Ca_V\alpha_2\delta$-1+HA-FMRP (104±12%, n=8, *P*=0.840). The intensity of the signal was expressed relative to that of the GFP band in each experiment. Means±s.e.m.; one-way ANOVA.

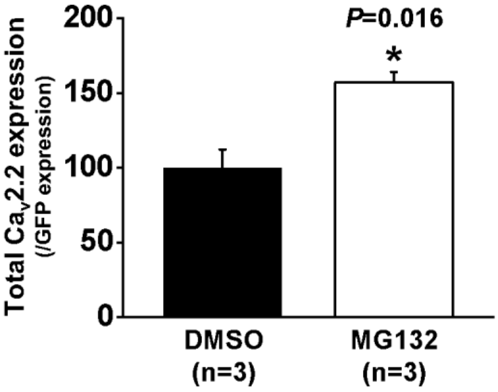

Supplementary Figure 3. Effect of proteasomal inhibition by MG132 on expression of Cav2.2 in tsA-201 cells. Expression of total $Ca_V2.2$ in tsA-201 cells expressing $Ca_V2.2$ either treated with DMSO (black bar, 100±12%, n=3) or MG132 (open bar, 156±7%, n=3, P=0.016). Means±s.e.m., *P<0.05; one-way ANOVA.

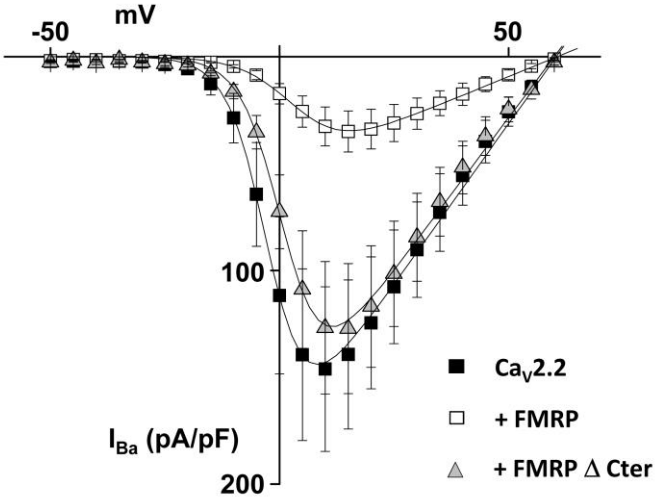

Supplementary Figure 4. Effect of C-terminal truncation of FMRP on $Ca_V2.2$ currents.

Current-voltage relationship from tsA-201 cells transfected with $Ca_V2.2$ (filled squares, n=7), $Ca_V2.2$+FMRP (open squares, n=5) and $Ca_V2.2$+FMRP lacking its C-terminal domain (FMRPΔCter; grey triangles, n=7). At +10mV, average peak current densities were -146±38pA/pF, -33±9pA/pF and -126±31pA/pF for $Ca_V2.2$, $Ca_V2.2$+FMRP and $Ca_V2.2$+FMRPΔCter, respectively. $Ca_V2.2$ *vs* FMRPΔCter: *P*=0.757; FMRP *vs* FMRPΔCter: *P*=0.032. Recordings were made from tsA-201 cells co-transfected with $Ca_V\beta1b$ and $Ca_V\alpha_2\delta$-1 subunits. The charge carrier was 1mM Ba^{2+}.

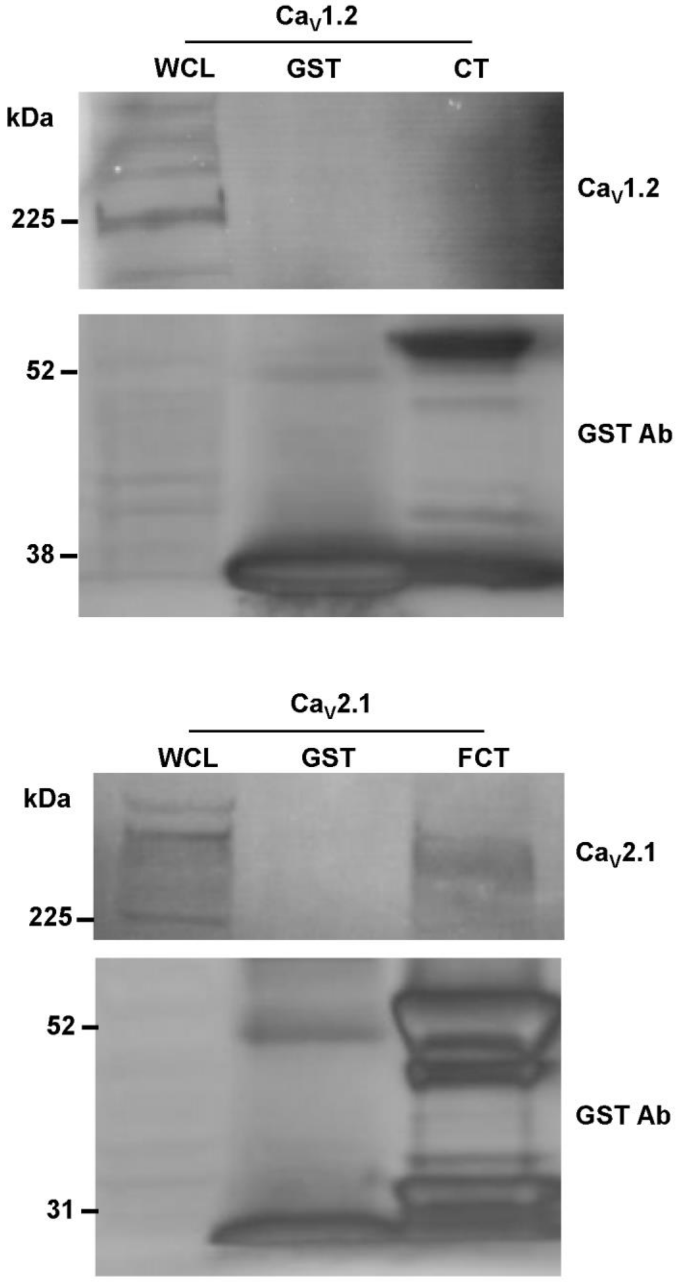

Supplementary Figure 5. FMRP also interacts with Ca$_V$2.1 but not Ca$_V$1.2

Western blots of pull-down assays reveal that Ca$_V$1.2 (upper panel), but not Ca$_V$2.1 (lower panel), expressed in tsA-201 cells (together with Ca$_V\alpha_2\delta$-1 and Ca$_V\beta$1b) bound FMRP C-terminus (FCT). Input represents 5% of protein input included in the assay. Bottom western blots show the amount of GST-tagged protein used. Immunoblots were performed using Ca$_V$1.2, Ca$_V$2.1 and GST Abs. Representative of 3 experiments.

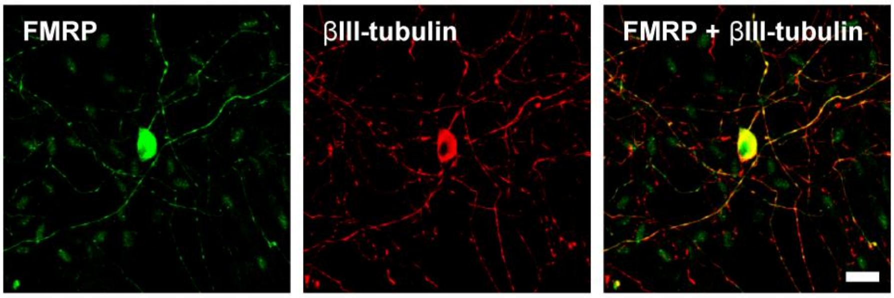

Supplementary Figure 6. FMRP is expressed in somata and processes of DRG neurons.

Representative confocal images showing expression of FMRP in soma and processes of cultured DRG neuron. DRG neurons were immunostained for FMRP (left panel, green) and βIII-tubulin (middle panel, red). The merged image is shown of the right panel. Scale bar, 20μm.

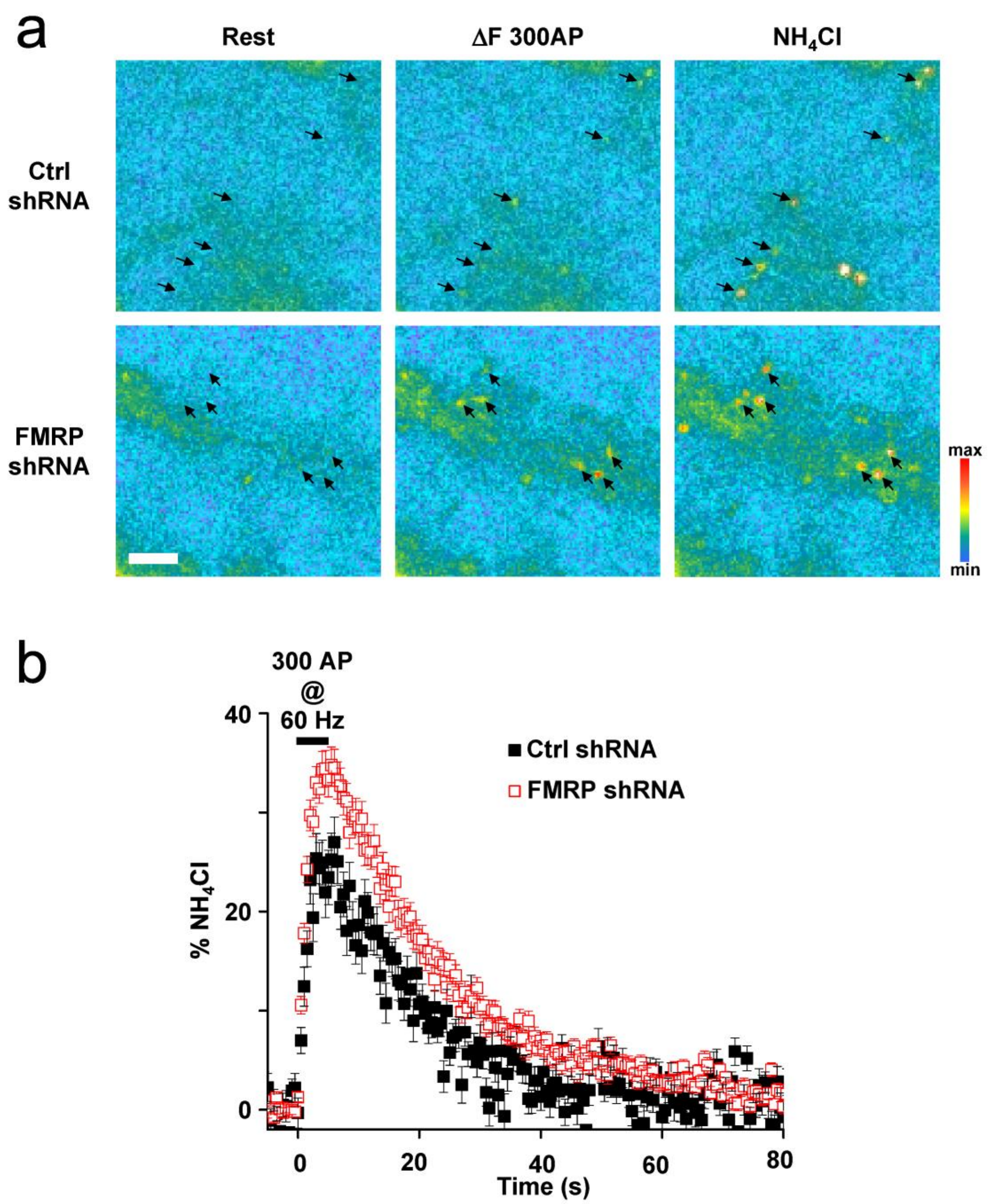

Supplementary Figure 7. FMRP increase vesicle release in hippocampal neurons.

(a) Representative images showing the fluorescence change (ΔF) of vGlut1-pHluorin in presynaptic terminals of hippocampal neurons transfected with Ctrl shRNA (Top panels) or FMRP shRNA (bottom panels) in response to electrical stimulation. Left panels: at rest; middle panels: after 300 action potentials (AP) at 60Hz; right panels: after a brief application of NH_4Cl. Responsive terminals are indicated by the black arrows.Pseudo-color scale is shown to the right (min, max: minimum and maximum fluorescence intensity). Scale bar 10μm. (b) Average traces of vGlut1-pHluorin response to 300 AP at 60Hz from presynaptic terminals of hippocampal neurons transfected with Ctrl shRNA (black filled squares) or FMRP shRNA (open red squares). Fluorescence intensities were normalized to the peak of a brief application of NH_4Cl. Peak fluorescence after 5 s stimulation was 25.3±2.7% for Ctrl shRNA (n=34) and 34.8±1.8% for FMRP shRNA (n=86, $P<0.0047$). Means±s.e.m.; one-way ANOVA.

Supplementary Figure 8. Full gel related to indicated Figures. Red boxed regions correspond to the portion used in the figures.

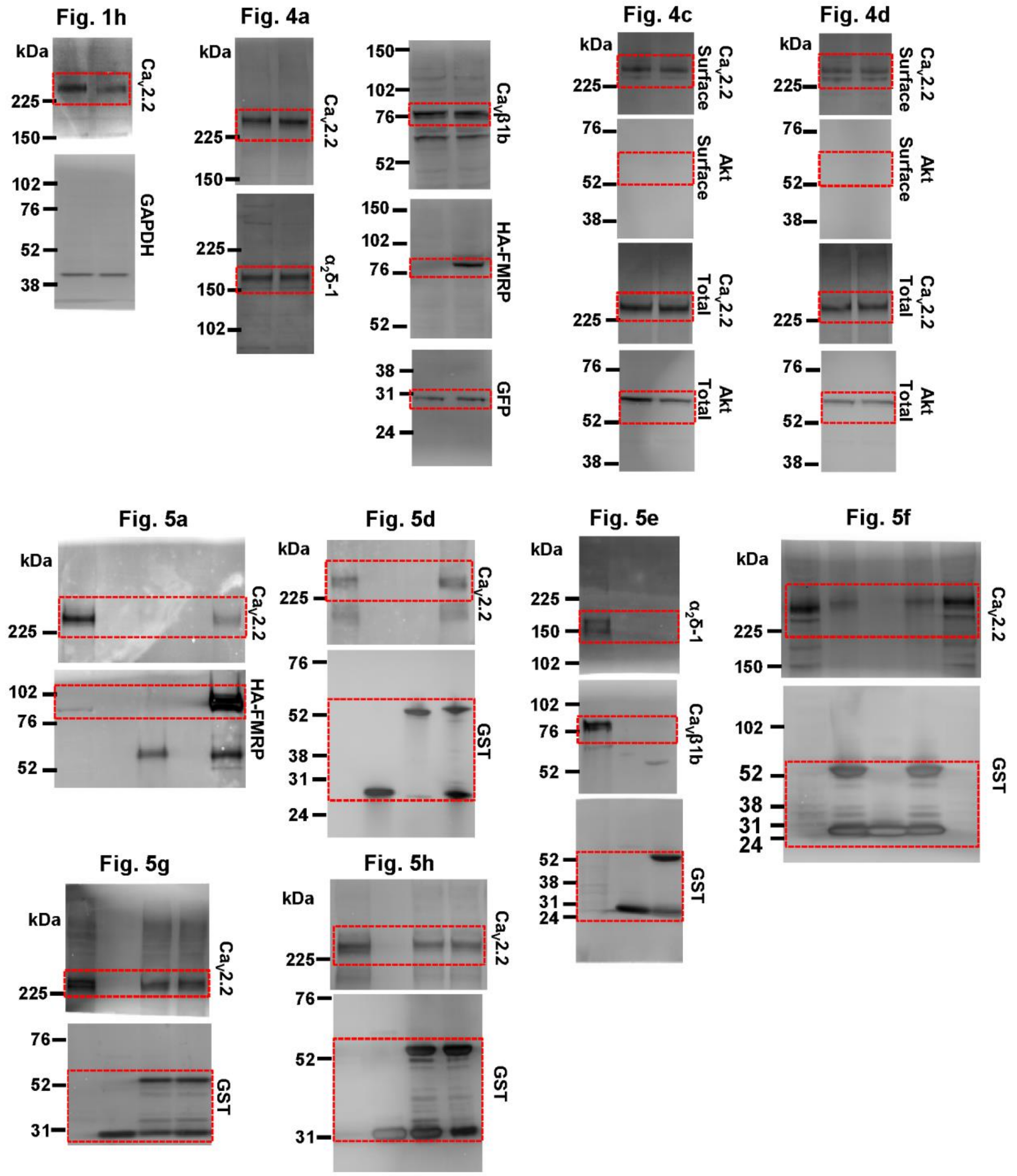

Fig. 6a

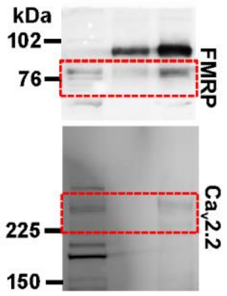

Fig. 7b

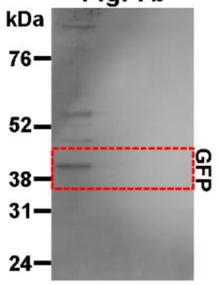

Fig. 7e

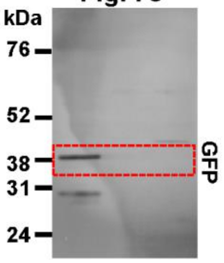

Fig. 6b

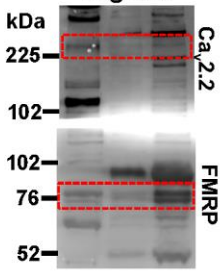

Fig. 7c

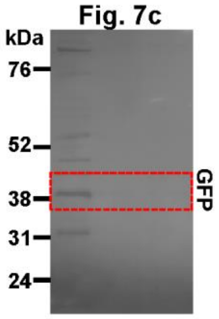

Fig. 7g

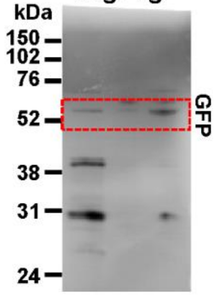

Fig. 6c

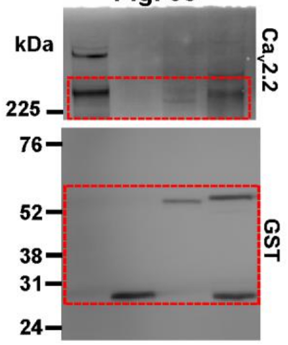

Fig. 7d

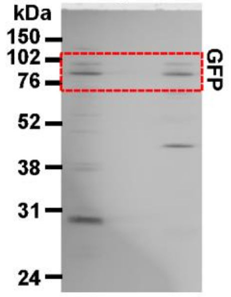

Fig. 7f

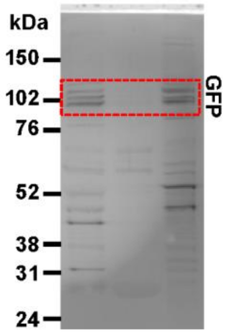

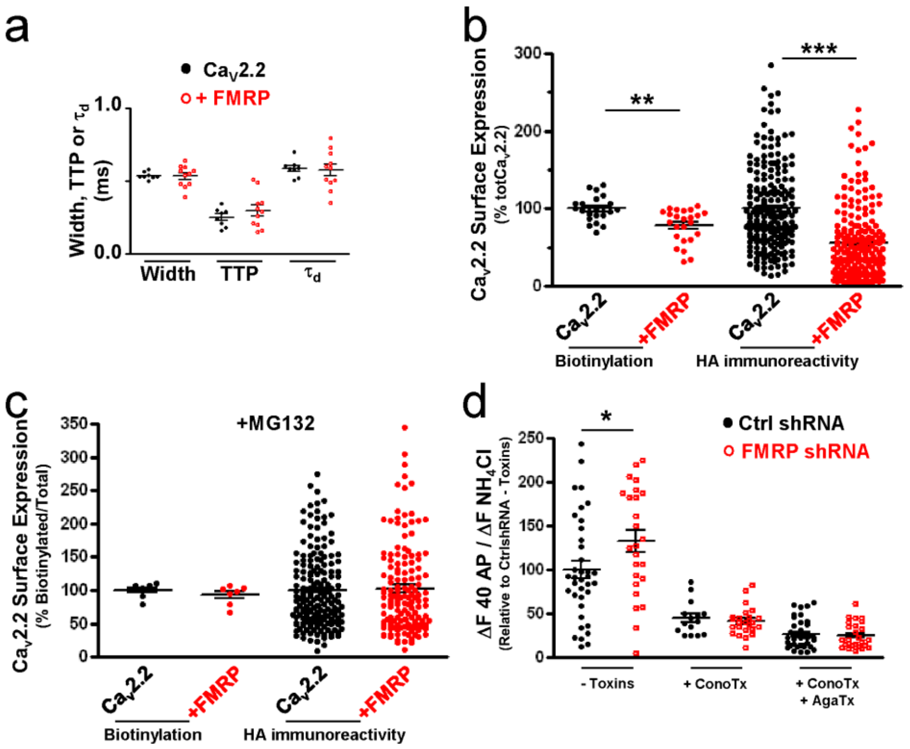

Supplementary Figure 9. Dot plot graphs for data presented in Fig. 3f, Fig. 4g, Fig. 4h and Fig. 8e.

(a) Figure 3f as a dot plot graph. Statistical comparison of peak ON-gating current properties for Ca_V2.2 (filled circles, n=8) and Ca_V2.2+FMRP (red open circles, n=11). Parameters of gating current kinetics measured were: Width (peak width at 50% of the maximum gating current), 0.54±0.01ms for Ca_V2.2 and 0.53±0.07ms for Ca_V2.2+FMRP (*P*=0.9); TTP (time-to-peak), 0.25±0.06ms for Ca_V2.2 and 0.29±0.12ms for Ca_V2.2+FMRP (*P*=0.3); and τ_d (mono-exponential decay time constant), 0.58±0.05ms for Ca_V2.2 and 0.57±0.13ms for Ca_V2.2+FMRP (*P*=0.6). Black bars represent the means ± s.e.m.

(b) Figure 4g as a dot plot graph. Surface expression of HA-Ca_V2.2 in tsA-201 cells expressing HA-Ca_V2.2 (black circles) or HA-Ca_V2.2+FMRP (red circles), together with accessory subunits, $Ca_V\beta$1b and $Ca_V\alpha_2\delta$-1. Left: the surface-biotinylated Ca_V2.2 band was corrected for the intensity of the total Ca_V2.2 in each experiment (Ca_V2.2: 100.0±3.2%, n=16; Ca_V2.2+FMRP: 71.3±7.8%, n=17, *P*=0.001). Right: HA surface immunoreactivity was normalized to the Ca_V2.2 condition following correction for the background noise (Ca_V2.2: 100.0±3.8%, n=200 cells; Ca_V2.2+FMRP: 56.1±3.3%, n=198 cells, *P*<0.00001). ***P*<0.01; ****P*<0.001. Black bars represent the means ± s.e.m.

(c) Figure 4h as a dot plot graph. Surface expression of Ca_V2.2 in tsA-201 cells expressing Ca_V2.2 (black circles) or Ca_V2.2+FMRP (red circles), following treatment with MG132 (n=5 for each condition). Left: surface-biotinylated Ca_V2.2 (Ca_V2.2: 100.0±4.2%, n=7; Ca_V2.2+FMRP: 92.9±5.6%, n=7, *P*=0.33). Right: HA immunoreactivity (Ca_V2.2: 100.0±4.3%, n=174 cells; Ca_V2.2+FMRP: 102.4±5.6%, n=142 cells, *P*=0.72). Black bars represent the means ± s.e.m.

(d) Figure 8e as dot plot graph. Normalized vGlut1-pHluorin responses to 40 AP at 10Hz from presynaptic terminals of DRG neurons transfected with Ctrl shRNA (black circles, 100±10.6%, n=38) or FMRP shRNA (red circles, 137.0±12.6%, n=25, *P*=0.027). ω-conotoxin GVIA (1μM) reduces Ctrl shRNA and FMRP shRNA responses to a similar level (44.7±4.9%, n=15 and 41.6±3.3 %, n=24, respectively). ω-conotoxin GVIA (1μM) and ω-agatoxin IVA (300nM) application reduces further the responses: Ctrl shRNA=17.3±3.2%, n=38, and FMRP shRNA=18.1±5.0%, n=27. **P*<0.05. Black bars represent the means ± s.e.m.
